# Safety of integrated mass drug administration of azithromycin, albendazole and ivermectin versus standard treatment regimens: a cluster-randomised trial in Ethiopia

**DOI:** 10.1016/j.eclinm.2023.101984

**Published:** 2023-04-27

**Authors:** Scott McPherson, Getinet Tafese, Temesgen Tafese, Sinknesh Wolde Behaksra, Hiwot Solomon, Birhanu Oljira, Hirpa Miecha, Kaleab A. Debebe, Biruck Kebede, Teshome Gebre, Fikreab Kebede, Fikre Seife, Fentahun Tadesse, Belete Mammo, Abraham Aseffa, Anthony W. Solomon, David C.W. Mabey, Michael Marks, Endalamaw Gadisa

**Affiliations:** a Clinical Research Department, Faculty of Infectious and Tropical Diseases, London School of Hygiene & Tropical Medicine, London, UK; b International Trachoma Initiative, Decatur, GA, USA; c Department of Control of Neglected Tropical Diseases, World Health Organization, Geneva, Switzerland; d Hospital for Tropical Diseases, University College London Hospital, London, UK; e Division of Infection and Immunity, University College London, London, UK; f Armauer Hansen Research Institute, Addis Ababa, Ethiopia; g Disease Prevention and Control Directorate, Ministry of Health, Addis Ababa, Ethiopia; h Oromia Regional Health Bureau, Addis Ababa, Ethiopia; i RTI International, Research Triangle Park, NC, USA

**Keywords:** Co-administration, Integration, Lymphatic filariasis, Trachoma, Onchocerciasis, Soil transmitted helminths, Safety

## Abstract

**Background:**

Neglected Tropical Disease (NTD) programs require separate and distinct drug regimens for treatment. This has required countries to undertake multiple independent mass drug administration (MDA) programmes, each targeting one or more diseases. The possibility of safely combining different drug regimens together in one MDA may offer several advantages to national programs. We conducted a study to assess the safety of combining ivermectin, albendazole and azithromycin in one integrated MDA.

**Methods:**

We conducted an open-label, non-inferiority cluster-randomised trial comparing the frequency of adverse events in communities receiving co-administered ivermectin, albendazole and azithromycin to that in communities given albendazole and ivermectin MDA followed by azithromycin MDA after a two-week interval. The study took place in 58 gares (small administrative units) across two kebeles (sub-districts) in Kofele woreda (district) in the Oromia region of Ethiopia. We randomly assigned 29 gares to the combined treatment arm and 29 gares to the control arm. The study team revisited all individuals within 48 h and actively collected data on the occurrence of adverse events using a dedicated questionnaire and a pre-specified list of adverse events. The study team followed the same process in the control arm for the azithromycin distribution and again after the ivermectin plus albendazole distribution. Following this initial active surveillance, passive surveillance was undertaken for one week after the first visit. The primary outcome was the frequency of adverse events occurring following MDA. The study team determined that the safety of the combined MDA would be non-inferior to that of separate MDAs if the upper limit of the two-sided CI for the difference in rates was equal to or lower than 5%. The trial was registered with ClinicalTrials.gov, NCT03570814.

**Findings:**

The study took place from December 2021 to January 2022. The combined MDA arm consisted of 7292 individuals who were eligible to participate, of whom 7068 received all three medications. The separate MDA arm consisted of 6219 eligible individuals of whom 6211 received ivermectin and albendazole and 4611 received azithromycin two weeks later. Overall, adverse events were reported by 197 (1.2%) of individuals. The most commonly reported adverse events included headache, gastrointestinal disturbance and dizziness. There were no serious adverse events in either arm. The cluster-level mean frequency of reported adverse events varied markedly between clusters, ranging from 0.1 to 10.4%. The cluster-level mean frequency of adverse events was 1.4% in the combined MDA arm and 1.2% following ivermectin and albendazole MDA (absolute difference 0.2%, 95% confidence interval [CI] −0.6% to +1.1%). This met the pre-defined 1.5% non-inferiority margin. For the combined MDA comparison to the stand-alone azithromycin MDA the absolute difference was −0.4% (1.4 versus 1.8%, 95% CI −0.8 to +1.5) which also met the pre-specified non-inferiority margin.

**Interpretation:**

This study is the largest of its kind to date and demonstrates that the safety of combined MDA of azithromycin, ivermectin and albendazole is non-inferior to the safety of ivermectin-plus-albendazole MDA then azithromycin MDA conducted separately although we may not have been powered to detect very small differences between arms. Co-administration of these three medicines is safe and feasible in this setting and allows national programs to develop new strategies for integrated MDA programs.

**Funding:**

Ivermectin (Mectizan) was donated by the Mectizan Donation Program, albendazole was donated by GlaxoSmithKline, and azithromycin (Zithromax®) was donated by Pfizer via the International Trachoma Initiative (ITI). The trial was funded by ITI using operational research funds from the 10.13039/100000865Bill and Melinda Gates Foundation.

**Research in context**

**Evidence before this study**

We searched PubMed for studies on the safety of co-administration of ivermectin, albendazole and azithromycin published between January 1, 1990, and February 5, 2023. Two small pharmacokinetic studies had established that there was limited evidence for drug–drug interactions between these agents. A third study additionally included diethylcarbamazine and equally found limited evidence of drug–drug interactions. One field study had been conducted which found no evidence of an increase in adverse events when the drugs were administered together rather than separately. However, this study involved only four clusters and was underpowered to provide adequate data on the safety of co-administration. A large cluster randomised trial in Papua New Guinea had examined the safety of co-administration. However, this trial had included the use of diethylcarbmazine and had altered the mechanism used for recording adverse events during the study period, making interpretation more complex.

**Added value of this study**

This is the largest published trial assessing the safety of co-administration of ivermectin, albendazole and azithromycin. We randomly allocated more than 12,000 individuals to either separate or combined administration. We observed no serious adverse events in either arm and no increase in the overall number of adverse events using the co-administration strategy.

**Implications of all the available evidence**

Based on the evidence from both pharmacokinetic and field-studies the evidence suggests that co-administration is a feasible and safe strategy in areas co-endemic for multiple relevant neglected tropical diseases. Switching to a co-administration strategy should support more rapid progress to the 2030 targets for the eradication, elimination and control of neglected tropical diseases.

## Introduction

The group of 20 poverty related diseases called neglected tropical diseases (NTDs), affecting marginalized people in the tropics and elsewhere, represent not only health challenges but also a significant social and economic burden to affected communities and health systems. With an estimated 200,000 associated deaths and 19 million disability adjusted life years (DALYs) lost annually, NTDs cost the equivalent of billions of United States dollars each year in direct health costs, loss of productivity and reduced socioeconomic and educational attainment, reinforcing the vicious cycle of poverty. Globally, over 1.7 billion people need prevention and treatment for at least one of these diseases, every year.^1^

Mass drug administration (MDA) is an intervention strategy in which medicines are offered to every member of a targeted population within a defined area, regardless of whether specific individuals are affected by the infection or disease of interest. NTDs that can be controlled in part through MDA include lymphatic filariasis (LF), onchocerciasis, schistosomiasis, soil-transmitted helminthiases, and trachoma. Each of these is treated with specific medicines or medicine combinations delivered within MDA campaigns. Supported by large scale drug donations, MDA for NTDs is often delivered at low cost and is commonly considered one of the most cost-effective interventions in global health. Traditionally, however, control of diseases requiring separate drug regimens has required countries to undertake multiple distinct MDA programmes, each targeting one or more diseases.

The recent NTD road map 2021–2030 has set global targets and milestones to prevent, control, eliminate and eradicate multiple NTDs by 2030. The platform from which we will achieve these targets is built on three pillars: (i) accelerating programmatic action, (ii) intensifying cross-cutting approaches, and (iii) changing operating models and culture to facilitate country ownership.^2^ To solidify the first and second pillars, national programmes are exploring ways to integrate different aspects of many current disease-specific interventions. This should help to achieve disease-specific 2030 targets, while easing the strain on country health systems and adapting to shifting availability of financial resources. One strategy is to explore co-administration of a wider range of medicines in combined MDA regimens. Various combinations of medicines have been previously used in integrated MDA in multiple contexts, providing proof-of-concept and some experience of the viability of this strategy.^3^^,^^4^

A specific agent for which further data are required is azithromycin, given within MDA campaigns for elimination of trachoma as a public health problem, and also for the eradication of yaws. Azithromycin is donated to national trachoma programmes as Zithromax (Pfizer, New York, NY, USA) but current guidance provided with the donation is that administration is separated from MDA for other diseases. Practically, combining azithromycin MDA with ivermectin and albendazole, which target LF, onchocerciasis and soil-transmitted helminths, is likely to be advantageous, because of the considerable overlap in the populations affected by these diseases. A precondition for widespread adoption of integrated MDA is evidence for a lack of deleterious pharmacokinetic interactions and empirical data on the safety of co-administration. Pharmacokinetic studies have demonstrated that there is little to no drug–drug interaction between ivermectin and albendazole, or between those two drugs combined with azithromycin.^5^, ^6^, ^7^ Small-scale studies of co-administration of all three medicines have been performed, but whilst those studies showed an absence of severe adverse events, they were underpowered to inform programmatic decision making.^8^

Ethiopia is endemic for LF, onchocerciasis, schistosomiasis, soil-transmitted helminthiases and trachoma. Currently, Ethiopia, follows WHO-recommended MDA guidance for each disease in endemic districts. For trachoma, annual rounds of azithromycin mass drug administration are undertaken, with the duration of intervention dependent on the prevalence of trachomatous inflammation—follicular (TF) in children aged 1–9 years.^9^ To address onchocerciasis, WHO recommends annual or biannual ivermectin MDA for at least 14 rounds.^10^ LF is managed with annual MDA of ivermectin and albendazole for at least five years, which also treats soil-transmitted helminths except in places where ivermectin, diethylcarbamazine, and albendazole is indicated.^3^^,^^11^ Many districts in Ethiopia could in theory receive integrated MDA combining all three agents. We set out to evaluate the safety of co-administration of albendazole, ivermectin and azithromycin compared to standard MDA delivery in Ethiopia.

## Methods

### Study design

We undertook a cluster randomised, non-inferiority trial comparing the frequency of adverse events in communities receiving co-administered ivermectin, albendazole and azithromycin to that in communities given albendazole and ivermectin MDA followed by azithromycin MDA after a two-week interval. Findings are reported in line with the CONSORT guidelines.

The study received ethical approval from the National Research Ethics Review Committee (NRERC) of Ethiopia (reference 3-10/195/2018) and the London School of Hygiene & Tropical Medicine (reference 11,985). Based on low levels of literacy in the study population, permission to use verbal consent was specifically provided by the ethics committees. Study teams read the study consent form to all prospective participants and requested verbal permission to participate in the study. Individuals who declined to participate received treatment according to the usual MDA schedule. The study team established an independent Data Safety Monitoring Board to review any reported severe adverse events. Michael Marks and Scott McPherson have accessed and verified the data.

### Study setting and participants

**Fig. 1:**
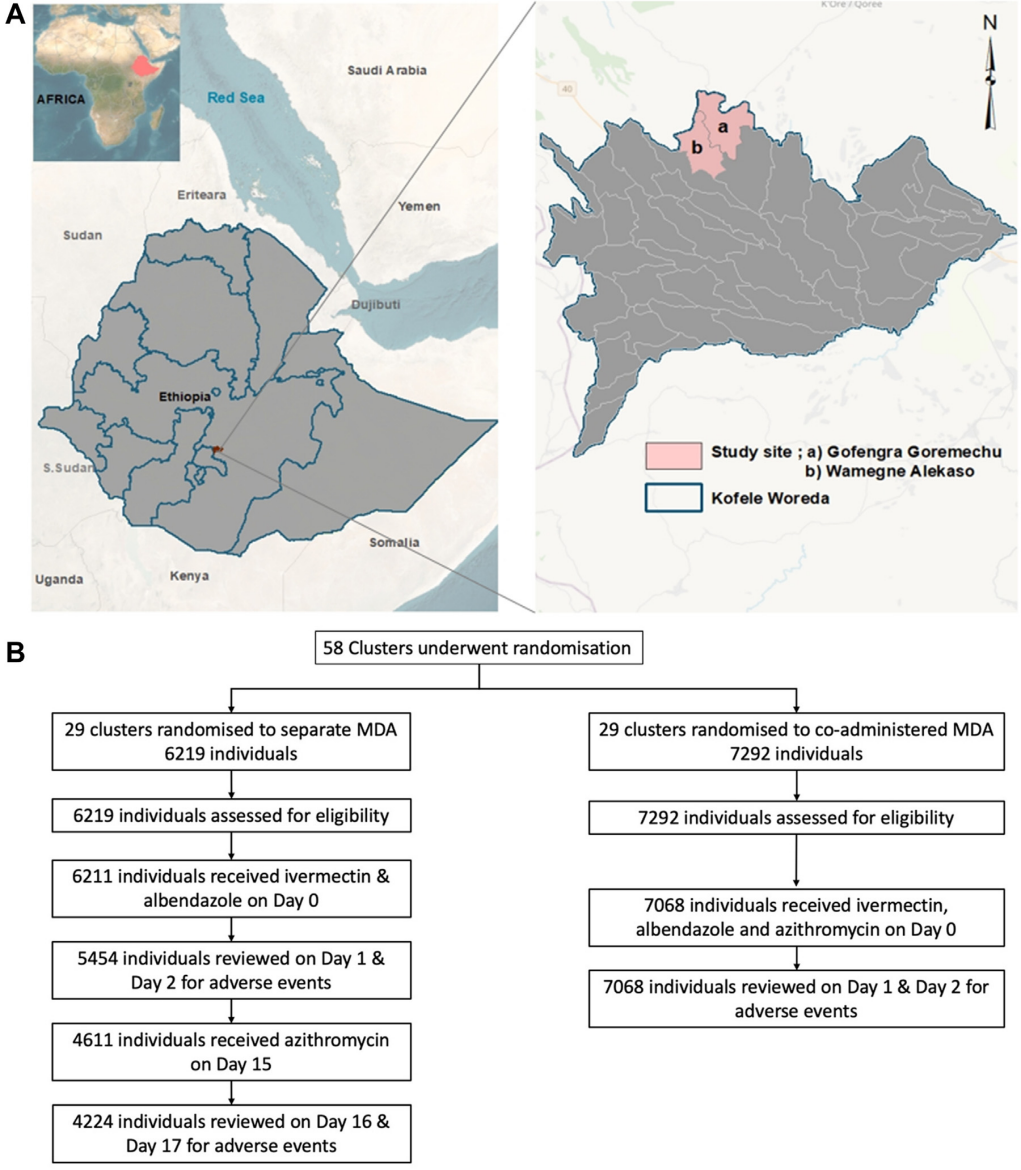
**Study location (A) and study profile (B)**. (A) The boundaries and names shown and the designations used on this map do not imply the expression of any opinion whatsoever on the part of the authors, or the institutions with which they are affiliated, concerning the legal status of any country, territory, city or area or of its authorities, or concerning the delimitation of its frontiers or boundaries. (B) D0/1/2/15/16/16 refer to study days. MDA, mass drug administration.

The study team reviewed a number of woredas (districts) which fit the co-endemicity profile specifications of the study but eventually selected Kofele woreda both for its absence of conflict as well as the support of zonal and woreda leadership. Kofele is one of 17 woredas in the West Arsi Zone in Oromia regional state (Fig. 1A). Based on the most recent data available prior to the trial, Kofele had a prevalence (estimated in 2017) of TF in children aged 1–9 years of 27.3% and a prevalence (estimated in 2020) of 1.5% for LF antigen in children aged 5–14 years. Prior to the trial, the woreda had received five annual rounds of MDA for LF and two rounds of MDA for trachoma delivered at separate timepoints.

Kofele woreda is divided into 18 kebeles. Each kebele is composed of a varying number of communities known as gares. Gares are defined by the government as a population of at least 350 individuals, but there is considerable variation in actual population. Within Kofele, we arbitrarily selected two kebeles, Gurmicho and Alkaso, to participate in the study. Prior to study commencement these kebeles had received two rounds of azithromycin MDA and five rounds of ivermectin and albendazole MDA. Combined, Gurmicho and Alkaso contained 58 gares.

In each community, individuals were eligible to receive treatment if they had been residing in the community for at least three months and were eligible to receive all three agents according to standard MDA criteria. Individuals were excluded from the trial if they were ineligible for any of the three study drugs, were unable to swallow tablets or declined to participate. Children aged under five years and pregnant or breastfeeding women were therefore considered ineligible as they could not receive ivermectin.

### Randomisation and blinding

The unit of randomisation was the gare. Randomisation was performed using the RANDNUM function in Microsoft Excel. Given that gares were randomly allocated from within the same woreda, had all had the same number of years of treatment with each medicine, and shared cultural and environmental factors, we assumed that there would be low variability of the outcome between clusters and therefore randomisation was not stratified by baseline cluster-level characteristics. The study was open label without blinding of participants or the field team.

### Interventions

Before the study began, the study team provided protocol specific training to the health extension workers in Kofele woreda. Training included an overview of the national NTD training manual followed by an in-depth explanation of the study protocol and how it differed from routine MDA procedure.

Gares were randomised to receive either combined MDA or separate MDA. In the combined MDA arm, recipients received, at a single timepoint, ivermectin, albendazole (400 mg) and azithromycin. The ivermectin dose was determined using 150 μg/kg dosage based on a standard height pole (90 cm–119 cm = 1 tablet; 120 cm–140 cm = 2 tablets; 141 cm–158 cm = 3 tablets; 158 cm and above = 4 tablets). Azithromycin dose was determined as follows: individuals ≥120 cm in height AND aged 7–15 years were offered azithromycin tablets of 250 mg each; the dose was either 3 or 4 tablets, determined by height. Individuals aged ≥15 years were given a full adult dose of 4 tablets, regardless of height. Individuals <120 cm in height or aged <7 years received azithromycin oral suspension instead of tablets. The separate MDA arm received ivermectin and albendazole at the first visit, followed by azithromycin two weeks later. All drugs were administered orally following standard WHO recommendations for directly observed treatment. The study team collected individual participant data using structured questionnaires. Data were collected using computer tablets into a study specific case reporting formula in REDCAP.

Following the co-administered MDA of all three drugs in the trial arm, the study team revisited all individuals within 48 h and actively collected data on the occurrence of adverse events using a dedicated questionnaire (Supplementary Appendix). The study team followed the same process in the control arm for the ivermectin plus albendazole distribution and again after the azithromycin distribution. Following this initial active surveillance, passive surveillance was undertaken for one week after the first visit following each MDA. We defined adverse and serious adverse events in line with Ethiopian national guidelines. In brief, adverse events were self-limiting or required minimal treatment. Adverse events were classified as mild, moderate or severe based on their impact on activities of daily living. Serious adverse events were those which required hospitalization, were life threatening or which resulted in death, disability or a congenital defect. All adverse events were followed up by the nurses until resolution.

### Statistical analysis

We estimated the anticipated rate of adverse events in the control arm based on previous studies.^8^, ^12^, ^13^ Forthe purposes of the study, we set the anticipated adverse event percentage at 7% for azithromycin. We assumed that this would vary between 6 and 8% across study clusters. With a non-inferiority margin of 1.5% and clusters of 190 individuals per cluster, we calculated we would require at least 36 gares in each study arm (72 gares in total) to have 90% power to meet our non-inferiority margin. The primary analysis was a per-protocol analysis on individuals who received the study drugs. For the primary analysis we calculated the cluster-level mean frequency of adverse events between arms and compared these with a T-test. For the secondary analysis we fitted a random-effects logistic regression model to compare the odds of adverse events between arms adjusting for age, gender and clustering at the level of the gare. Analysis was performed in R version 4.1.1. The trial was registered on clinicaltrials.gov (identifier: NCT03570814).

### Role of the funding source

Ivermectin (Mectizan) was donated by the Mectizan Donation Program, albendazole was donated by GlaxoSmithKline, and azithromycin (Zithromax®) was donated by Pfizer via the International Trachoma Initiative (ITI). The trial was funded by ITI using operational research funds from the Bill and Melinda Gates Foundation. Funders and donors had no role in data collection, data analyses, interpretation, or writing of the report.

## Results

Fieldwork took place from December 2021 to January 2022. A total of 58 gares were randomised 29 to integrated MDA and 29 to separate MDA (Fig. 1A). Overall, 13,511 people were assessed for study participation (Fig. 1B). The study population was 50.7% female and the median age was 14 years (IQR 10–19 years) (Table 1). The combined MDA arm consisted of 7292 individuals who were eligible to participate, of whom 7068 received all three medications. The separate MDA arm consisted of 6219 eligible individuals of whom 6211 received ivermectin and albendazole and 4611 received azithromycin two weeks later. Prior to MDA, 0.4% of individuals in the separate MDA arm and 0.4% of individuals in the combined MDA arm reported any baseline symptoms.

**Table 1:** Baseline demographics.

	Co-administered MDA (n = 7068)	Separate MDA: ivermectin plus albendazole (n = 6211)	Separate MDA: azithromcyin (n = 4611)
Median age, years (IQR)	14 (10–22)	13 (10–16)	10 (13–17)
Male sex, n (%)	3454 (48.9%)	3072 (49.5%)	2285 (49.6%)
Female sex, n (%)	3613 (51.1%)	3124 (50.3%)	2311 (50.1%)

**Fig. 2:**
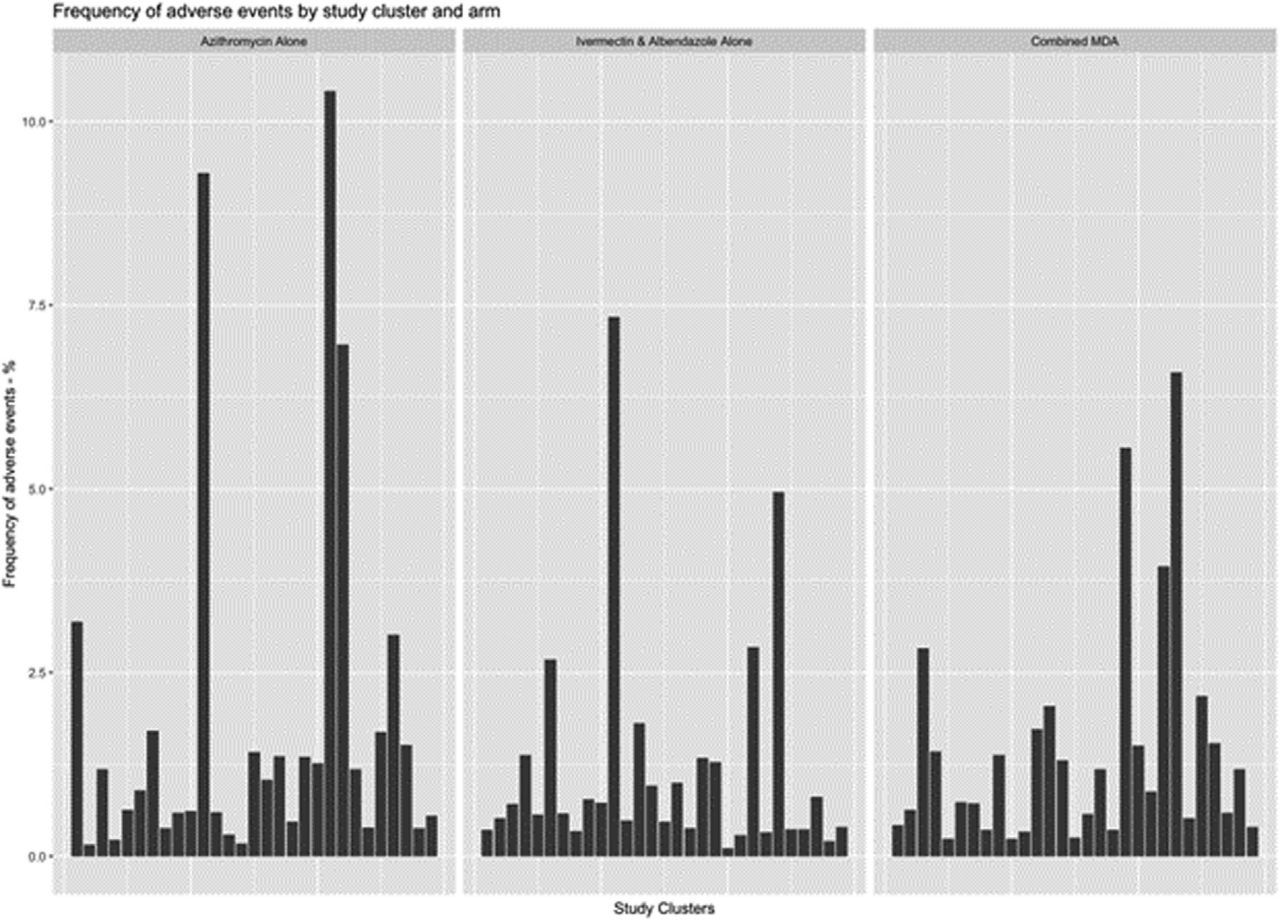
**Frequency of adverse events by cluster and study arm.** The proportion of individuals reporting at least one adverse event following either combined or separated MDA is shown for each cluster.

Overall, adverse events were reported by 197 (1.2%) of individuals. The most commonly reported adverse events included headache, gastrointestinal disturbance and dizziness. There were no serious adverse events in either arm. The cluster-level mean frequency of reported adverse events varied markedly between clusters (Fig. 2), ranging from 0.1 to 10.4%. The cluster-level mean frequency of adverse events was 1.4% in the combined MDA arm and 1.2% following ivermectin and albendazole MDA (absolute difference 0.2%, 95% confidence interval [CI] −0.6% to +1.1%). This met the pre-defined 1.5% non-inferiority margin. For the combined MDA comparison to the stand-alone azithromycin MDA the absolute difference was −0.4% (1.4 versus 1.8%, 95% CI −0.8 to +1.5) which also met the pre-specified non-inferiority margin.

We fitted a random effects model to assess the relationship between study arm and the risk of adverse events after adjusting for age and gender. The risk of adverse events was the same in individuals who received combined MDA, and individuals who received ivermectin-albendazole alone (aOR 1.28, 95% CI 0.6–2.8, p = 0.5). Similarly, the risk of adverse events was the same in individuals who received combined MDA and those who received azithromycin alone (aOR, 1.2 95% CI 0.6–2.3, p = 0.6). Neither age nor gender were associated with frequency of adverse events (Table 2).

**Table 2:** Adverse events comparing co-administered MDA with both ivermectin & albendazole MDA and with azithromycin MDA.

	Integrated MDA versus albendazole plus ivermectin			Integrated MDA versus azithromycin		
	aOR	95% CI	p	aOR	95% CI	p
Integrated MDA	1.2	0.6–2.8	0.5	1.2	0.62–2.3	0.6
Age	0.99	0.97–1.0	0.14	0.99	0.97–1.0	0.2
Sex	1.0	0.68–1.60	0.9	0.78	0.52–1.2	0.2

## Discussion

In this study, the largest of its kind to date, we demonstrate that the safety of combined MDA of azithromycin, ivermectin and albendazole is non-inferior to the safety of MDA of ivermectin plus albendazole, and azithromycin distribution conducted separately. Co-administration of these three medicines is safe and feasible in this setting. We also noted a reduced coverage of the second MDA in the communities randomised to the control arm, which might suggest that co-administration can help achieve both efficiencies and improved programmatic coverage. These are critical data for WHO and for national programs of NTD-endemic countries as they consider new strategies to save resources and accelerate progress towards NTD elimination and control targets. Taken together with the existing published data discussed below, which are similarly reassuring, it is our hope that these findings will pave the way for more widespread adoption of integrated MDA.

Co-administration could have significant programmatic impact, particularly in Ethiopia, where the study was conducted. As of the time of preparing this manuscript, Ethiopia had 441 woredas requiring antibiotic MDA for trachoma, 76 woredas requiring MDA for LF, and 243 woredas known to require MDA for onchocerciasis, of which 221 received semi-annual MDA. More than 50 of these woredas are co-endemic for trachoma and at least one other NTD treated with the regimens evaluated in this study and could therefore benefit from a co-administration strategy. Combining MDA could save money through the implementation of joint supply chains, health workforce training, drug administration and supervision, as has been observed in other triple drug therapy studies.^4^ MDA campaigns also require a significant time investment for the local health workforce, drawing personnel away from other duties, and multiple stand-alone MDA days may cause MDA fatigue within recipient communities. The combined MDA approach could concentrate MDA interventions for the needs and availability of the community, as has been suggested in MDA strengthening analyses published elsewhere.^14^

Our trial adds to the existing data on the safety of co-administration and importantly is the largest published study to date, overcoming a major shortcoming of previous studies. We noticed variation in the frequency of adverse events between communities, which might reflect chance or underlying cluster-level covariates; the frequency of adverse events in all clusters was within the range reported in other studies of MDA. Both randomised trial data in Mali and non-randomised data from the Solomon Islands and Colombia are concordant with our finding that co-administration is a safe and feasible strategy.^8^^,^^15^, ^16^, ^17^, ^18^ Our data are also in keeping with studies conducted in Papua New Guinea combining azithromycin with ivermectin, albendazole and diethylcarbamazine. A strength of the current study over this previous work is the use of consistent definitions and approaches to adverse event monitoring throughout the whole trial.^19^ Collectively, these data suggest that co-administration is an acceptable and safe approach to tackling co-endemic NTDs.

Our trial has some limitations. First, we randomised a smaller number of gares than originally planned. Whilst the average cluster size was slightly larger that anticipated we did reach our pre-planned sample size. As, on average, power in a cluster randomised trial is higher when there are a larger number of smaller clusters, this will have reduced our overall power to detect differences between arms. This is likely to be more marked for the comparison of combined MDA with azithromycin-only MDA, because a smaller number of individuals took part in the azithromycin-only MDA. As such we can not exclude the possibility that there remains a small difference between arms that we did not detect. Second, we focused on safety rather than effect on infection or disease, so we cannot assess whether co-administration increases or decreases efficacy. Previous pharmacokinetic studies have demonstrated^15^ little to no drug–drug interactions between these three medicines likely to affect efficacy.^5^^,^^7^^,^^20^ In addition, we noted a suggestion of increased coverage in the co-administration clusters which might be anticipated to result in improved efficacy. The selected study district had already received five rounds of MDA for LF and two for trachoma with reported high coverage. Therefore, the intensity of infections, particularly of helminths, was likely already at low levels and this may have contributed to the low burden of adverse events. Whilst this might explain the low absolute number of adverse events, we do not believe it would affect the comparison between arms and therefore our findings are likely also of relevance to districts which have not previously undertaken multiple rounds of MDA. Third, it was not possible to mask study participants or field teams and this might have affected reporting of adverse events. Finally, the original intention was that the study roll out using a central point distribution strategy in each cluster to mimic standard programmatic MDA. Due to the COVID-19 pandemic, we changed our MDA delivery to a house-to-house campaign. This forced a slower, more deliberate approach by the study teams. While we have established here that taking all three drugs concurrently is feasible, this might be affected by mode of distribution. However, we feel this is unlikely to have influenced the safety comparison in the study. We undertook a nested qualitative study to explore both healthcare worker and participant perspectives of the co-administration which will be reported separately.

WHO currently recommends five possible MDA medicine combinations but not azithromycin in any combination with ivermectin or albendazole.^21^ Health ministries, drug donation programmes, donors and implementing partners are showing significant interest in the implementation of integrated MDA programs to accelerate scale-up and drive greater efficiency. Our data provide the clearest evidence to date that such a strategy is safe and feasible. Adoption of integrated MDA may help accelerate progress to global NTD targets ahead of 2030.

## Contributors

SM, TG, AWS, DCWM, MM, EG designed the study. SM, GT, TT, SB, HS, BO, HM, KAD, BK, FK, FS, FT, BM, AA, EG ran the trial and collected data. SM, AWS, DCWM, MM, EG analysed the data. SM wrote the first draft of the manuscript. All authors revised the manuscript. SM and MM directly accessed and verified the underlying data.

## Data sharing statement

An anonymised copy of the data included in the analysis is available on request by email to Michael.Marks@lshtm.ac.uk.

## Declaration of interests

MM was supported by the International Trachoma Initiative to present findings of this work at the Trachoma Expert Committee meeting in December 2022. TG is an employee of the International Trachoma Initiative. AS is an employee of the World Health Organization. The views and opinions expressed in this paper are those of the authors and do not necessarily reflect the views or opinions of these organizations.

## Appendix A Supplementary data

Current Co-Administration Protocol

CONSORT Checklist
