# Corrigendum to “Safety of integrated mass drug administration of azithromycin, albendazole and ivermectin versus standard treatment regimens: a cluster-randomised trial in Ethiopia”

**DOI:** 10.1016/j.eclinm.2023.102120

**Published:** 2023-07-20

**Authors:** Scott McPherson, Getinet Tafese, Temesgen Tafese, Sinknesh Wolde Behaksra, Hiwot Solomon, Birhanu Oljira, Hirpa Miecha, Kaleab A. Debebe, Biruck Kebede, Teshome Gebre, Fikreab Kebede, Fikre Seife, Fentahun Tadesse, Belete Mammo, Abraham Aseffa, Anthony W. Solomon, David C.W. Mabey, Michael Marks, Endalamaw Gadisa

**Affiliations:** a Faculty of Infectious and Tropical Diseases, Clinical Research Department, London School of Hygiene & Tropical Medicine, London, UK; b International Trachoma Initiative, Decatur, GA, USA; c Department of Control of Neglected Tropical Diseases, World Health Organization, Geneva, Switzerland; d Hospital for Tropical Diseases, University College London Hospital, London, UK; e Division of Infection and Immunity, University College London, London, UK; f Armauer Hansen Research Institute, Addis Ababa, Ethiopia; g Ministry of Health, Addis Ababa, Ethiopia; h Oromia Regional Health Bureau, Addis Ababa, Ethiopia; i RTI International, Research Triangle Park, NC, USA

The authors regret that the published version of this article contained errors in the methods section of the abstract and in Table 1 as corrigendum.

**Table 1:** Baseline demographics.

	Co-administered MDA (n = 7068)	Separate MDA: ivermectin plus albendazole (n = 6211)	Separate MDA: azithromcyin (n = 4611)
Median age, years (IQR)	14 (10–22)	13 (10–16)	13 (10–17)
Male sex, n (%)	3454 (48.9%)	3072 (49.5%)	2285 (49.6%)
Female sex, n (%)	3613 (51.1%)	3124 (50.3%)	2311 (50.1%)

In the methods section of the abstract, the non-inferiority margin was written as 5%. This was a typographical error. We have provided the correct version, which is 1.5%, as stated in the main body of the paper and the supplementary appendix. The sentence now reads: “The study team determined that the safety of the combined MDA would be non-inferior to that of separate MDAs if the upper limit of the two-sided CI for the difference in rates was equal to or lower than 1.5%”.

In Table 1, the median age and IQR for the azithromycin group was mistakenly written as: 10 (13–17). This was a typographical error. We have provided the corrected version, which is: 13 (10–17).

These corrections do not change the interpretation or conclusions of the paper. The authors would like to apologise for any inconvenience caused.

