# Supplementary material for: Safety of integrated mass drug administration of azithromycin, albendazole and ivermectin versus standard treatment regimens: a cluster-randomised trial in Ethiopia
Source: eClinicalMedicine. 2023 Apr 27;59:101984. doi: 10.1016/j.eclinm.2023.101984 (PMC10154979; doi:10.1016/j.eclinm.2023.101984)
Supplement: Current Co-Administration Protocol [file mmc1.pdf]

Safety of the co-administration of azithromycin, albendazole and ivermectin versus standard treatment regimens during mass drug administration (MDA) in Ethiopia: a cluster-randomized trial

Scott McPherson

Gadisa Endalamaw

Version 1: 5/16/2018

London School of Hygiene & Tropical Medicine is the main research sponsor for this study. For further information regarding the sponsorship conditions, please contact the Research Governance and Integrity Office:

London School of Hygiene & Tropical Medicine

Keppel Street

London WC1E 7HT

Tel: +44 207 927 2626

Email: RGIO@lshtm.ac.uk

London School of Hygiene & Tropical Medicine holds Public Liability ("negligent harm") and Clinical Trial ("non-negligent harm") insurance policies which apply to this trial.

This protocol describes the "Homesha" study and provides information about procedures for entering participants. The protocol should not be used as a guide for the treatment of other participants; every care was taken in its drafting, but corrections or amendments may be necessary. These will be circulated to investigators in the study, but centres entering participants for the first time are advised to contact the trials centre to confirm they have the most recent version.

Problems relating to this trial should be referred, in the first instance, to the study coordination centre.

This trial will adhere to the principles outlined in the International Conference on Harmonisation Good Clinical Practice (ICH GCP) guidelines, protocol and all applicable local regulations.

Study Proposal

Chief Investigators

Scott McPherson[1]

Gadisa Endamalaw[2]

Co-Investigators

Taye T. Balche[2]

David Mabey[1]

Michael Marks[1]

David McCleod[1]

Anthony Solomon[1]

Biruck Kebede[3]

Nebiyu Negessu[3]

Kebede Deribe[5]

Abraham Aseffa[2]

Paul Emerson[6]

[1] London School of Hygiene & Tropical Medicine, London, UK

[2] Armauer Hansen Research Institute

[3] Ethiopia Federal Ministry of Health

[4] World Health Organization, Geneva, Switzerland

[5] Wellcome Trust

[6] **International Trachoma Initiatives**

Table of Contents

ACRONYMS LIST **5**

ABSTRACT **7**

STUDY PROTOCOL SUMMARY **8**

1. Disease Profile of Lymphatic Filariasis (LF), Onchocerciasis (OV), Soil Transmitted Helminths (STH) and Trachoma in Ethiopia **10**

1.1. Lymphatic Filariasis (LF): General Information 10
Lymphatic Filariasis in Ethiopia 10
1.2. Onchocerciasis (OV): General Information 11
Onchocerciasis in Ethiopia 11
1.3. Soil Transmitted Helminths (STH)- General Information 12
STH in Ethiopia 12
1.4. Trachoma- General Information 13
Trachoma in Ethiopia 13

2. Modes of Action of the study drugs and their administered dosage **13**

1.5. Azithromycin 13
1.6. Ivermectin 13
1.7. Albendazole 14

3. Programmatic Advantages of Co-Administration of Azithromycin, IVM, and ALB in Ethiopia: **15**

3.1. Prevalence of TF in 1-9 year olds > 10% and LF or LF/OV co-endemic: 15
3.2. Prevalence of TF in 1-9 year olds > 5% and LF or LF/OV co-endemic: 15
3.3. Prevalence of TF in 1-9 year olds > 10% and co-endemic with STH 20%-50% only (not LF or OV endemic): 15
3.4. Prevalence of TF in 1-9 year olds > 10% and co-endemic with STH above 50% (not LF or OV endemic): 15

4. Feasibility of Triple Drug Co-Administration Based on Existing Data **17**

4.1. Pharmacokinetic Data on Combined Drug Interaction of azithromycin, ivermectin and albendazole 17
1.8. Past studies involving permutations of azithromycin, IVM and ALB combined 17

5. Study Aim **18**

5.1. Specific objectives 18

6. Description of the Study Area .... Error! Bookmark not defined.

6.1. Homesha woreda .... **Error! Bookmark not defined.**

7. Participant Selection Criteria **19**

7.1. Inclusion Criteria 20
7.2. Exclusion Criteria 20

8. Co-administration MDA Strategy **21**

8.1. Statistical Methods 21

8.2. Awareness Creation, Community Mobilization Mechanism, and Training 22
8.3 Co-Administration Team Allocation 23
8.4 Recording of any AE/SAEs 23
8.5 Analysis Plan of Reported AEs/SAEs 25
8.6 Co-administration Team Composition 26
8.7 Co-administration Team Composition 27
8.8 Co-Administration Schedule 27
8.9 Data Collection and Statistical Analysis 32

9 Ethical Clearance 33

10 Timetable for Study Completion 34

11 Secondary Outcomes 35

SUPPLEMENTARY APPENDICES 41

Appendix 1: Partner Organizations participating in the study (largely copied from Institution Websites): 41

Appendix 2: Case recording forms 43

Appendix 3: Treatment form 46

Appendix 4: AES Surveillance form 47

Appendix 5: SAEs recording form 53

Appendix 6: Participant Information Sheet for Co-administration Participants 57

ACRONYMS LIST

ALB	Albendazole
APOC	African Programme for Onchocerciasis Control
CNTD	Centre for Neglected Tropical Diseases, Liverpool School of Tropical Medicine
CY	Calendar Year
DFID	Department for International Development (U.K.)
DSA	Disease-Specific Assessment
EOEEAC	Ethiopia Onchocerciasis Elimination Expert Advisory Committee
EPHI	Ethiopian Public Health Institute
F and E	Facial Cleanliness and Environmental Improvement (part of the SAFE strategy)
FMOH	Federal Ministry of Health
FPSU	Filariasis Programmes Support Unit, Liverpool School of Tropical Medicine (formerly known as CNTD)
GTMP	Global Trachoma Mapping Project
HDA	Health Development Army
HEW	Health Extension Worker
ICT	Immunochromatographic Test
IEC	Information, Education and Communication
ITI	International Trachoma Initiative
IVM	Ivermectin
LF	Lymphatic Filariasis
LFTW	Light For The World
M&E	Monitoring and Evaluation
MDA	Mass Drug Administration
MEB	Mebendazole
MMDP	Morbidity Management and Disability Prevention Program
MOH	Ministry of Health
MOU	Memorandum of Understanding
NGO	Nongovernmental Organization
NTD	Neglected Tropical Disease
OEPA	Onchocerciasis Elimination Program for the Americas
OV	Onchocerciasis
PCR	Polymerase Chain Reaction
PC	Preventive Chemotherapy
PHCU	Primary Health Care Unit
PZQ	Praziquantel
REMO	Rapid Epidemiological Mapping of Onchocerciasis
RHB	Regional Health Bureau
RTI	RTI International
SAC	School-Aged Children
SAE	Serious Adverse Events
SAFE	Surgery-Antibiotics-Facial cleanliness-Environmental improvements
SCH	Schistosomiasis
SCI	Schistosomiasis Control Initiative
SNNPR	Southern Nations, Nationalities, and People's Region
STH	Soil-Transmitted Helminths
TAS	Transmission Assessment Survey
TF	Trachomatous Inflammation–Follicular
TIPAC	Tool for Integrated Planning and Costing

TOT	Training of Trainers
TT	Trachomatous Trichiasis
UIG	Ultimate Intervention Goal
USAID	U.S. Agency for International Development
WASH	Water, Sanitation, and Hygiene
WHO	World Health Organization
ZTH	Zithromax®

ABSTRACT

Ethiopia has one of the largest burdens of neglected tropical diseases in the world. The Ethiopian Federal Ministry of Health has set 2020 as the year for the elimination of three of these NTDs: blinding trachoma, lymphatic filariasis, and onchocerciasis. The FMOH has also set 2025 for the elimination of soil transmitted helminths as a public health problem within the country. In order to achieve these goals, Ethiopia must address its endemic communities through years of mass drug administration (MDA) of azithromycin for trachoma, ivermectin for onchocerciasis, ivermectin and albendazole for lymphatic filariasis, and either mebendazole or albendazole for soil transmitted helminths. Currently, WHO guidelines do not condone the combination of azithromycin with any of the other aforementioned drugs during MDA, instead requiring two distinct MDAs separated by several days. This requirement has significant cost implications and human resource demands for national programs and requires a greater investment of time for recipient communities in co-endemic woredas. Pharmacokinetic data as well as some small co-implementation studies suggest that triple drug administration of azithromycin, ivermectin and albendazole is safe. This study seeks to establish the safety profile, assess the community acceptance, and weigh the economic advantages of this three-agent MDA through a non-inferiority randomized control trial. Using a cohort event monitoring design which relies upon both passive and active pharmacovigilance, this study will take place in one district in Oromia *region. Within this district, the study will target a recipient population of at least 16,000 people. The primary outcome will be to demonstrate the safety of the triple-drug administration as measured by incidence of AEs/SAEs over a one month period following the MDA. Secondary outcomes of the study will include a qualitative analysis of the perceptions of the beneficiaries receiving the co-administration especially surrounding pill burden and the change in MDA distribution schedule; a qualitative analysis of the perceptions of the co-administration strategy by the local health workforce as a means of PC-NTD drug delivery; a cost analysis comparing existing MDA unit costs from woredas within the regions already on a standard MDA schedule with that of the co-administered woredas; and a literature/data review across 19 different countries including Ethiopia which will analyse the possible benefits of co-administration from an economic and epidemiological standpoint when compared with current trends towards achieving 2020 NTD control and elimination goals on a global scale.*

STUDY PROTOCOL SUMMARY

History of revision		
1	5/16/2018	Conditionally approved by LSHTM, waiting the approval from Ethiopia
2	09/08/2018	Approved by AHRI/ALERT Ethics Committee

Title	Safety of the co-administration of azithromycin, albendazole and ivermectin versus standard treatment regimens during mass drug administration (MDA) in Ethiopia: a cluster-randomized trial
Objective	To establish the safety profile, assess the community acceptance, and weigh the economic advantages of a three-agent MDA combining azithromycin, albendazole (Alb), and ivermectin (Ivm) in comparison to the standard MDA regiment which separates the administration of azithromycin from the dually administered ivermectin and albendazole by a period of two weeks.
Design	A cluster-randomized non-inferiority trial to assess the safety, local acceptance, and economic advantages of combined MDA with azithromycin, albendazole, and ivermectin in one district in Oromia, Ethiopia. Within this district, a study group of 8,000 people will receive the triple drug co-administration. A control group of 8,000 people within the same district will receive the standard MDA treatment beginning with Ivm/Alb followed two weeks later with azithromycin. The study will randomly sort sub-district communities *(Gotes)* into the trial arm and the control arm. The study will compare the “baseline” of adverse events (AEs) and severe adverse events (SAEs) between the two arms to determine if the triple drug therapy is not inferior to the standard treatment.
Study Duration	6 months
Interventions	All residents of the selected communities will be invited to participate in the co-administered drug treatment strategy. Consenting/assenting residents of the villages will be screened as per the inclusion and exclusion criteria. Dosage and treatment age groups will follow WHO protocols for lymphatic filariasis, onchocerciasis, STH,[1] and trachoma[2]. Study enrolment will continue until the sample size is attained.
Number of subjects	A total of 16,000 people: 8,000 people in the trial arm and 8,000 in the study arm. If there is no increase in adverse events in the trial arm, triple-drug therapy will be used to treat the remaining 4-5,000 people in the district.
Population	All individuals living in the selected communities who agree to participate. See below for more information on exclusion criteria.

[1] WHO: Preventive chemotherapy in human helminthiasis
http://apps.who.int/iris/bitstream/10665/43545/1/9241547103_eng.pdf

[2] WHO: Trachoma Control- A guide for trachoma program managers
http://apps.who.int/iris/bitstream/10665/43405/1/9241546905_eng.pdf

1. Disease Profile of Lymphatic Filariasis (LF), Onchocerciasis (OV), Soil Transmitted Helminths (STH) and Trachoma in Ethiopia1

1.1. Lymphatic Filariasis (LF): General Information

Lymphatic filariasis is a vector borne disease caused by three different nematodes: *Bruges Malaya, burgee tamari and Wucheria bancrofti*, of which *Wucheria bancrofti* is the most common (90% of infections). Though LF is usually not fatal, the morbidity manifested by the disease makes it the the second-leading parasitic cause of disability in the world.[2] More than 15 million people globally are affected by lymphedema caused by LF, which mostly commonly manifests as swelling of the legs, but it can also occur in the arms, breasts and genitals. In addition to lymphedema, more than 25 million males suffer from uro-genital swelling caused by hydrocele.[3] Acute adenolymphangitis (ADL), known to cause painful, episodic febrile onsets, is also common and is caused by bacterial infections related to the lymphedema.[4]

Lymphatic filariasis occurs when infective larva (microfilaria in the L3 stage), spread by *anopheles, culex, and aedes* mosquito vectors, enter into the human lymphatic system and develop into adults. The adults will most commonly take residence in the lymphatic nodes blocking the lymphatic drainage system, thereby causing a pooling of lymph which can cause extensive damage and the aforementioned manifestations of morbidity. Female adult worms produce offspring which migrate to the blood and lymph channels with nocturnal periodicity for the optimal opportunity of infecting a new mosquito vector. To treat endemic communities, the WHO recommends five rounds of IVM/ALB before conducting an impact assessment.

Lymphatic Filariasis in Ethiopia

Starting in June 2013, Ethiopia targeted 57 woreda suspected to be endemic for LF through a nationwide initiative led by the Ethiopia Public Health Institute (EPHI) and the FMOH NTD team. The 2013 mapping was conducted using current WHO guidelines for initial LF assessments: In each implementation unit, two sites were selected based on the high likelihood of ongoing transmission, and in each site, a convenience sample of 100 adults aged 15 years or older was tested for antigenemia by ICT. During this 2013 mapping initiative, podoconiois mapping was also conducted by identifying woredas as endemic for podoconiosis if lymphedema cases were found but exhibited negative ICT results.[5]

As stated in the revised National Master Plan (2016–2020)[6] and in accordance with the WHO Global LF-Elimination Strategy, the FMOH is targeting LF for elimination by 2020. In compliance with *Lymphatic Filariasis: A Manual for National Elimination Programs,* the national program uses an MDA strategy combining IVM and ALB in entire at-risk populations. MDA coverage must be at least 65% of the total population in an endemic area for at least five years before conducting transmission assessment surveys (TAS) to determine whether MDA can be stopped. In the 45 LF-endemic woredas that are co-endemic with OV, ALB can be added to the existing IVM MDA. Currently, the triple drug administration of ALB, IVM, and PZQ is not used in practice, although this strategy may be considered by the FMOH in some co-endemic areas after one to two years of separate treatments, according to WHO guidelines. In areas targeted for LF MDA, school aged children (SAC) are not specifically targeted with a separate MDA for STH unless the woreda has prevalence >50%, and bi-annual treatment is required. It is important to note that *Loa* is not endemic in Ethiopia and, thus, does not present a barrier to using IVM.[7]

1.2. Onchocerciasis (OV): General Information

Onchocerciasis is the second leading infectious cause of blindness, behind only trachoma. More than 120 million are at risk for OV, more than 96% of which reside in sub-Saharan Africa.[9] In addition to causing blindness, OV can cause painful skin disfiguration as well as musculoskeletal pain. Onchocerciasis is caused by the parasite *Onchocerca volvus* which is spread by the *Simulium* fly vector. The flies breed along fast flowing streams and rivers, hence the common name "river blindness". *Onchocerca volvus* enters a human host while a female infected *Simulium* fly takes its blood meal. The OV larvae develop into adults in the subcutaneous tissue where they will remain for 10-12 years in pronounced nodules. A female worm can produce 1300-1900 microfilaria a day for 9 years. It is these microfilaria which navigate through the dermal layers and to the eye to cause OV-related morbidity. To address OV in an infected population, the WHO recommends annual or biannual treatment with ivermectin (further detailed below).[8-11]

Onchocerciasis in Ethiopia

In 2013, Ethiopia declared that the country's National NTD Master Plan was shifting from OV control to OV elimination. OV elimination is defined by the WHO and FMOH as follows[9]

- *Interventions have reduced O. volvulus infection and transmission below the point where the parasite population is believed to be irreversibly moving to its extinction.*
- *Interventions have been stopped.*
- *Post-intervention surveillance for an appropriate period has demonstrated no recrudescence of transmission to a level suggesting recovery of the O. volvulus population.*
- *Additional surveillance is still necessary for the timely detection of recurrent infection.*

In 2014, national and international experts formed the Ethiopia Onchocerciasis Elimination Expert Advisory Committee (EOEEAC) to help guide the FMOH in implementing this strategic shift. In October 2014, the committee held its inaugural meeting which focused on creating the national OV elimination guidelines. The creation of the document was based on the WHO Geneva 2001-approved guidelines and the 2013 WHO/NTD Strategic and Technical Advisory Group draft guidelines, with consideration of the experiences of the Onchocerciasis Elimination Program for the Americas (OEPA), and APOC. The guidelines propose several strategies, including bi-annual MDA, transmission zone mapping, and targeted vector control. The overarching theme for interventions described by the guidelines is that each OV-endemic area requires a tailored approach rather than the one-size-fits-all interventions practiced by the APOC model.

Based on the successes of in Uganda and Sudan, the guidelines recommend bi-annual MDA with IVM as the main strategy for interrupting transmission. The FMOH currently endorses bi-annual treatment for newly endemic areas that are IVM naïve or any annual treatment area that is not on track to end MDA in 2020. The elimination guidelines stipulate that moving woredas from an annual to a bi-annual treatment schedule should be dictated by the following indicators:

- *The positive skin snip rate among adults in any community is >2%.*
- *Skin snip-positive children <10 years of age are found in any community.*
- *The OV-16 rates in children <10 years exceed >0.1% (95% confidence interval).*
- *The PCR infectivity in flies exceeds >1/2,000 (95% confidence interval).*

- *The seasonal transmission potential (as calculated by PoolScreen®) exceeds 20 L3/person/year (95% confidence interval).*[10]

As of May 2017, 153 of the 188 of the woredas endemic for OV are on a bi-annual treatment schedule (Figure 1). The guidelines stipulate that impact assessments will be conducted in these woredas after five years of bi-annual treatments.
Whether or not OV mapping is "complete" is technically difficult to determine. For a long time, the FMOH and APOC have assumed that only the western part of the country would have OV because the vast majority of fast-flowing rivers are found in this area.[11] However, recent mapping in arid countries found OV in areas previously thought to be environmentally unsuitable. Although understanding the OV situation in the eastern part of the country is important, currently, the FMOH is not suggesting that a full mapping initiative is required. Instead, in 2017, the FMOH plans to conduct a targeted entomological evaluation to determine the occurrence of OV transmission before confirming that eastern Ethiopia represents a "gap" in mapping.

1.3. Soil Transmitted Helminths (STH)- General Information

The WHO estimates that more than 2 billion people in the world are infected with the classification of intestinal worms that make soil-transmitted helminths.[12] Roundworm (*Ascaris lumbricoides*), whipworm (*Trichuris trichiura*) and hookworm (*Necator americanus* and *Ancylostomaduodenale)* comprise the three STH nematodes and are spread through human feces either through hand/mouth contact with infected soil (roundworm and whipworm) or through the soles of bare feet (hookworm). Global morbidity caused by STH infection has been estimated at 5.18 million DALYs.[13]

STH in Ethiopia

Though not stated in the WHO NTD roadmap, Ethiopia has taken the initiative to eliminate STH (and Schistsomiasis (SCH) though not relevant to this study) so that it will no longer represent a public health problem by 2025. This goal will require the repeated treatment of at least 75% of SAC (enrolled and non-enrolled) in Ethiopia. According to the National STH/SCH Action Plan, the long-term goals associated with this control program are as follows:

- *Eliminate STH-related morbidity in children by 2020*
- *Reduce the proportion of individuals harboring heavy infection with STH by 60%*
- *Ensure that treatment coverage is expanded to pre-school children in the future*

STH infections are distributed very widely throughout the country, and more than 62 million people are estimated to be living in the 476 STH-endemic woredas. It is important to note that final mapping results have not yet been made available for parts of Afar, Somalia, and Amhara regions. The FMOH plans to treat hypo-endemic woredas for SCH every two years rather than every three years because of the logistical constraints involved in successfully implementing a program with such a long interval between treatments. The FMOH will most likely distribute mebendazole with ANY SCH MDA—regardless of whether the woreda in question is above the 20% threshold for STH—to maximize logistical and cost efficiency of SCH treatments. While any woreda above 20% STH prevalence is currently targeted for school-based MDA (ages 5-14), the FMOH also targets any community above 50% STH prevalence with twice-a-year, community-wide Alb MDA as per WHO guidelines. I

1.4. Trachoma- General Information

Trachoma is caused by *Chlamydia trachomatis* and is spread by direct personal contact, fomites, and by flies (specifically *Musca sorbens*). It is the number one infectious cause of blindness in the world.[14] After many years of repeated infections, scars in the upper eyelid can cause the eyelashes to invert and press against the eye, resulting in *trachomatous trichiasis*. This stage of trachoma can lead to eventual corneal opacity through scarring of the cornea.[15] The WHO has set the ultimate intervention goal of reducing the prevalence of follicular trachoma (TF) throughout the world to lower than 5%. To accomplish this, varying rounds of mass drug administration with azithromycin, dependent on the prevalence of TF in children 1-9, followed by an impact assessment are recommended: 5-9.9% prevalence requires one year of MDA, 10-29.9% prevalence requires 3 years of MDA, 30%-49.9 requires 5 rounds of MDA and areas with greater than or equal to 50% prevalence require *at least* 7 rounds of MDA.

Trachoma in Ethiopia

The FMOH is following the 2020 elimination goals set forth by WHO which state that clinical signs of active trachoma (TF) should be found in less than 5% of children aged 1–9 years, and TT cases, unknown to the health system, occur in less than 1 per 1,000 people living in a woreda. With more than 82.8 million people currently requiring intervention through MDA and an estimated TT backlog of 880,317, achieving these goals by 2020 represents a great challenge for Ethiopia.

Largely in thanks the Global Trachoma Mapping Project (GTMP), trachoma surveys throughout the country are now complete, except in some zones affected by the insecurity in Somali region. The results of these mapping efforts revealed that 575 woredas (68% of the woredas in the country) have trachomatous inflammation–follicular (TF) prevalence at or above 10%. However, with the availability of Pfizer-donated Zithromax for one round of MDA for woredas with baseline TF prevalence of 5%–9.9%, 93 additional woredas will require support for a single round of MDA and the subsequent impact assessment. This will bring the total woredas targeted for MDA to 668.

2. Modes of Action of the study drugs and their administered dosage

Azithromycin

After ingestion, azithromycin spread quickly and widely through the body. While albendazole and ivermectin are primarily absorbed by the plasma in the human body, azithromycin is found in much higher levels (as much as 50 times) in the tissue. It is also absorbed by immune cells such as phagocytes which help to transport it to sources of infection. Azithromycin is intracellularly active which help explains its efficacy against bacteria such as *C. trachomatis*.[16]

For mass drug administration in Ethiopia, Zithromax is given as a single observed dose, determined by dose pole as per WHO recommendation. Everyone 5 years and older receives their dose in 250 mg tablets while children between 6 months and 5 years old will receive syrup (200 mg per 5 ml). Individuals aged 6 months and under as well as pregnant women are offered topical tetracycline. [17]

1.6. Ivermectin

Ivermectin's deleterious effect on nematodes such as *onchocercus volvus* is still not fully understood. The class of broad-spectrum, anti-parasitic drug that ivermectin is a part of is attracted to the chloride ion channels that are found in the muscle and nerve cells of nematodes and other invertebrates. It is believed that once ivermectin moves into these channels, it essentially blocks the ability of the cells to function, thereby causing paralysis of the nematode and eventual death. [3] However, in culture, ivermectin does not have a direct effect on microfilaria, suggesting that the human immune system may also play a role. In other studies, ivermectin has been shown to reduce the ability of nematode cells to produce a particular protein which may usually help disguise it from the host immune system.[18] Interestingly, ivermectin does not threaten the nervous system of mammals because of its inability to cross the fully-developed blood-brain barrier and enter the central nervous system, which is why the drug is safe for humans.

In terms of controlling or eliminating OV infection in a population, many years of MDA are necessary because ivermectin is useful against the larval forms *Onchocerca volvus as a* microfilaricide, but has little to no effect against the adult forms, which live for years within nodes of the skin where the drug cannot reach. The half-life of ivermectin once metabolized is approximately 12-36 hours and is excreted in the faeces for up to 12 days.[19]

For mass drug administration in Ethiopia, those who are 5 years and older (due to need for a "fully developed" blood/brain barrier) will be given a single observed dose in tablet form (3 mg each), the size of which is determined by dose pole as per WHO recommendation.[20]

1.7. Albendazole

Once a patient has swallowed the single-dose, 400 mg tablet, albendazole is metabolized primarily into albendazole sulfoxide which has a half-life within the body of eight and a half hours. Albendazole blocks the ability of the cytoskeletons in helminthic cells by attacking the microtubules and impairing the cell's ability to absorb glucose.[21] Albendazole works against both the adult and larval stages of helminths. In terms of the focal diseases of this study, albendazole is effective against LF when combined with IVM and the three soil transmitted helminths: roundworm (*Ascaris lumbricoides*), the whipworm (*Trichuris trichiura*) and hookworm (*Necator americanus* and *Ancylostomaduodenale)*. For mass drug administration in Ethiopia, Albendazole is prescribed as a single oral dose of 400mg.

[3] https://www.merck.com/product/usa/pi_circulars/s/stromectol/stromectol_pi.pdf

3. Programmatic Advantages of Co-Administration of Azithromycin, IVM, and ALB in Ethiopia:

It is important to establish the possible benefits of triple drug co-administration within a population before embarking upon a study of this nature. Within Ethiopia, the different permutations of trachoma, LF, STH, and IVM that justify the programmatic advantage logic of co-administration are:

3.1. Prevalence of TF in 1-9 year olds > 10% and LF or LF/OV co-endemic:

This category captures the **30 woredas** that are currently targeted for trachoma MDA as well as LF MDA or LF/OV MDA. Woredas co-endemic with OV are of particular importance in terms of a co-administration as Ethiopia has adopted an OV elimination strategy which includes twice a year MDA in all OV-endemic areas. This added round of MDA increases costs by 40-50% per year. If trachoma MDA can be integrated with OV MDA, then this could potentially negate the cost of a second round of OV MDA.

3.2. Prevalence of TF in 1-9 year olds > 5% and LF or LF/OV co-endemic:

Ethiopia adopted the targeting of MDA-naïve woredas that have TF prevalence between 5 to 10% as part of the national strategy in 2015. If the safety of co-administration is established, the FMOH can address **40 woredas** inexpensively by integrating them into the existing LF or LF/OV MDA rounds.

3.3. Prevalence of TF in 1-9 year olds > 10% and co-endemic with STH 20%-50% only (not LF or OV endemic):

If trachoma and STH (SAC-only) endemic woredas are treated with the same MDA, this may be a useful mechanism for capturing non-enrolled school children, a major challenge for the national program, in **96 woredas**.

3.4. Prevalence of TF in 1-9 year olds > 10% and co-endemic with STH above 50% (not LF or OV endemic):

Woredas above a 50% prevalence of STH require two rounds of MDA as well as MDA for high risk groups which are defined by the FMOH as the rest of the community population. Pairing this second round with trachoma MDA would negate the costs of the additional round in **116 woredas**. Conversely, using this population-wide STH treatment as a launching board for trachoma MDA would address more than half the remaining woredas in the country currently not under a trachoma MDA schedule due to lack of funding.

Table 1: Co-Endemic Woredas which could be addressed through various permutations of Azithromycin, ALB, and IVM

	**Prevalence of TF in 1-9 year olds ≥ 10% and LF co-endemic only**	**Prevalence of TF in 1-9 year olds ≥ 5% and LF co-endemic only**	**Prevalence of TF in 1-9 year olds ≥ 10%** and LF/OV co-endemic	**Prevalence of TF in 1-9 year olds ≥ 5%** and LF/OV co-endemic	**Prevalence of TF in 1-9 year olds ≥ 10%** and OV co-endemic only	**Prevalence of TF in 1-9 year olds ≥ 5%** and OV co-endemic only	**Prevalence of TF in 1-9 year olds ≥ 10%** and co-endemic with STH 20%-50% only (not LF or OV endemic)	**Prevalence of TF in 1-9 year olds ≥ 10%** and co-endemic with STH above 50% (not LF or OV endemic)	**Prevalence of TF in 1-9 year olds ≥ 5%** and co-endemic with STH 20%-50% only (not LF or OV endemic)	**Prevalence of TF in 1-9 year olds ≥ 5%** and co-endemic with STH above 50% (not LF or OV endemic)
Number of Woredas in Ethiopia*	81	92	30	40	114	146	96	116	100	126

*Average population per woreda is 100,000

4. Feasibility of Triple Drug Co-Administration Based on Existing Data

4.1. Pharmacokinetic Data on Combined Drug Interaction of azithromycin, ivermectin and albendazole

Pharmacokinetic studies have demonstrated that there is little to no drug-to-drug interaction between ivermectin and albendazole[22]. Mass co-administration of both drugs to treat LF has also occurred with no reported serious adverse events related to drug to drug interactions among large populations for many years. The question lies in the safety of adding azithromycin to the albendazole/ivermectin combination. In a randomized, three-way crossover pharmacokinetic study on the interaction between azithromycin, ivermectin and albendazole, 18 volunteers were administered 500 mg of azithromycin, 400 mg of albendazole, and a dose proportional to body weight of ivermectin concurrently. The results of the study found the interactions between albendazole and azithromycin to be small enough to be of little clinical importance. The total drug exposure over time for ivermectin increased by 31% among the volunteers.[23] This trend of higher peak blood levels of ivermectin when co-administered with azithromycin has been demonstrated within pharmacokinetic models to be well within safety ranges. [24] The study author goes on to say in conclusion that the population pharmacokinetic model analyses "support further study of co-administration of azithromycin with the widely used agents ivermectin and albendazole, under field conditions".[23]

1.8. Past studies involving permutations of azithromycin, IVM and ALB combined

Mali: This was a study in four randomly assigned villages (N=3, 011) endemic for trachoma and LF. Two villages were randomized for "co-administering ivermectin, albendazole, and azithromycin" and two villages were given the standard treatment of one round of OV/LF MDA followed one week later by a round of azithromycin MDA. In the study, the overall reported rates of any adverse event were similar: 18.7% (281/1501) in the co-administration arm and 15.8% (239/1510) in the standard treatment arm. No serious adverse event was reported. However, the study was too small to justify a definitive recommendation.[25]

Solomon Islands: a field trial of co-administration of azithromycin and ivermectin mass drug administration for scabies and trachoma (N=26,188) revealed no safety issues with combining azithromycin and ivermectin[26]. Follow up was completed for 21,931 participants (83.7%) and identified no serious adverse events. Adverse events were noted in 2.6% of participants across the entire study population; the most commonly reported adverse events were dizziness, abdominal pain and diarrhoea. This study did not, however, include the addition of albendazole.

Colombia: At the programmatic level, this work involved reaching remote trachoma and STH co-endemic populations. More than 305,005 people received albendazole and azithromycin together for three separate annual cycles (2012-14). The reported adverse events were headaches, dizziness and diarrhoea in 0.16% of those surveyed (7 out of 4,438 people). No serious adverse events were documented[27]. However, the findings are not statistically powered.

5. Study Aim

The aim of the study is to establish the safety profile, assess the community acceptance, and weigh the economic advantages of a three-agent MDA combining azithromycin, albendazole (Alb), and ivermectin (Ivm) in comparison to the standard MDA regiment which separates the administration of azithromycin from the dually administered ivermectin and albendazole by a period of two weeks.

5.1. Specific objectives

5.1.1. Primary Objective:

To establish the safety profile, assess the community acceptance, and weigh the economic advantages of a three-agent MDA combining azithromycin, albendazole (Alb), and ivermectin (Ivm) in comparison to the standard MDA regiment which separates the administration of azithromycin from the dually administered ivermectin and albendazole by a period of two weeks.

5.1.2. Secondary Objective:

- Understanding the perceptions of the beneficiaries receiving the co-administration of azithromycin, ivermectin, and albendazole especially surrounding pill burden and the change in MDA distribution schedule.

- Understanding the perceptions of the co-administration strategy by the local health workforce as a means of PC-NTD drug delivery.

- A cost analysis comparing existing MDA unit costs from woredas within the region already on a standard MDA schedule with that of the co-administered woredas.

- A literature/data review across 19 different countries including Ethiopia which will analyse the possible benefits of co-administration from an economic and epidemiological standpoint when compared with current trends towards achieving 2020 NTD control and elimination goals.

6. Description of the Study Area

The directly observed MDA by co-administration of azithromycin, IVM and ALB will take place within the woreda of Kofele in the West Arsi Zone of Oromia in region. This woreda has an estimated population of people. Within this population, the study requires a total of 13,680 individuals with 6, 840 in each arm. The study will target a population of 16,000 people which will allow a 15% cushion to factor in non-compliance and exclusionary criteria among the recipients. This woreda was selected due to the co-endemicityof LF and trachoma.

6.1. Kofele woreda

6.1.1. General information

Kofele is one of 17 woredas in West Arsi Zone in Oromia region of Ethiopia. West Arsi is bordered on
the south by the Sidama Region and the SNNPR, on the west by the SNNPR, on the northwest by East Shewa Zone, on the northeast by the Arsi Zone, and on the east by Bale Zone, and on the Southeast by Guji zone. The administrative center of the zone is in Shashemane. Kofele woreda is 27 KMs southeast from Shashemane, at an elevation of 2695 meters above sea level. Based on the 2014 E.C. estimate by FMoH the woreda has a population of 251,098.

6.1.2. Kofele NTD prevalence and treatment history

Kofele has a prevalence of 27.3% for trachoma folicullitis, 1.5% for LF. The woreda is currently under annual treatment schedule for LF and trachoma. This will be the fifth round of trachoma MDA conducted in the woreda.

7. Participant Selection Criteria

Standard FMoH criteria for individual agent MDA will be used to determine eligibility of individuals to receive each drug. Individuals eligible to receive all three agents, ‘co-administration’, will be enrolled in the study.

7.1. Inclusion Criteria

- Must have been residing in the community for at least three months;
- Eligible to receive all three agents according to standard MDA criteria;[28,29]

7.2. Exclusion Criteria

- Not eligible to receive one or more drugs according to standard MDA criteria;
- Less than 5 years of age (not eligible for ivermectin)**
- Pregnant women (azithromycin only, not eligible for albendazole and ivermectin)
- Lactating women (Only administered azithromycin and albendazole, not eligible for ivermectin);
- History of allergies to the drugs being studied (azithromycin, ivermectin, albendazole)
- Those who refused to be part of the study (will be addressed via the normal MDA schedule if they prefer with initial treatment with IVM/ALB and azithromycin treatment two weeks later)
- Residents who cannot swallow tablets;

***Note that patients that are not eligible for IVM, will receive azithromycin and albendazole. Patients that receive azithromycin and albendazole will be followed up through the same procedure as the triple drug therapy to try to track any AEs attributed to the two drug combination.*

8. Co-administration MDA Strategy

8.1. Statistical Methods

Clinical Randomized Controlled trials often try to demonstrate that one form of treatment is better than an existing treatment, typically known as a superiority trial. However, within this co-administration study, the goal is not to demonstrate the superiority of triple-drug administration over standard MDA in terms of efficaciousness. Rather, the study is will demonstrate whether administering all three drugs together is no worse, within a specified margin, than giving all three drugs via the standard distribution format.[30] The possible benefit of the co-administration trial will most likely lie in improved convenience and cost without any increase in negative outcomes, not necessarily in improved treatment results. For that reason, the study will use a non-inferiority trial model.

Within our standard arm, the study will deduce the average adverse event rate for azithromycin administered alone using information from previous trials, and it will assume an average adverse event rate for azithromycin, ivermectin and albendazole administered together. For azithromycin, there is a wide-range of reported adverse event rates. The aforementioned co-administration trial in Mali assumed an adverse event rate of 8%.[31] A randomized control trial using a single dose of azithromycin which took place in the United States reported adverse events at 17%[32]. A randomized control trial in Ethiopia reported that monitored adverse event rates after Zithromax MDA reported 4.9%-7.0% adverse events in children from 1-9 years old and 17.0% to 18.7% in persons ≥ 10 years of age.[33] Meanwhile, Pfizer labeling reports the following: "Side effects that occurred in patients on the single one-gram dosing regimen of Zithromax with a frequency of 1% or greater included diarrhea/loose stools (7%), nausea (5%), abdominal pain (5%), vomiting (2%), dyspepsia (1%) and vaginitis (1%)."[34] For the purposes of this study, we will set the adverse event percentage at 7% for azithromycin distribution. In terms of the adverse even rate during co-administration of all three drugs, adverse events related to IVM and ALB distribution are largely due to the death of microfilariae[35], particularly after the first round of MDA in a community. Given that this community has received several years of treatment with both drugs, we assume an acceptable increase in adverse events during co-administration will be 1.5% (Delta), or it will be acceptable for 8.5% of our intervention arm to experience an adverse event.

The study will require a power of 90% and a two-sided 95% confidence interval for the difference between rates. Given that gotes will be randomly selected from within the same woreda, have all had the same number of years of treatment with each drug, and share the same cultural and environmental factors, we will assume a low variability between each cluster. In case there are unforeseen differences between the clusters, we will include a variance between 6-8%. Conservatively assuming that each gote has a population of 190 individuals, the study will require at least 36 gotes in each study arm to reach 13,600. To take non-compliance, etc. into account, the study will randomize the 42 gotes into each study arm to reach 8,000 participants in each arm from the list of gotes in Kofele woreda into the control and study arms until each arm has 8,000 participants (to account for non-compliance, etc.). Gotes will be chosen via the RANDNUM function in excel. The randomization itself will be conducted via public randomization ceremonies.

Table 2: Assumption Summary

Adverse event prevalence	7% on average
Acceptable adverse event percentage during co-administration:	8.5%
Delta	1.5%
Variance	Adverse event prevalence between clusters will be 6-8%
Power	90%
CI	95%
Minimal Number of Gotes Required for each arm	36
Population per cluster	190 individuals per cluster (gote) on average
Total Population of Study	13,600 (Rounded to 16,000 for refusals/loss to follow up)

8.2. Awareness Creation, Community Mobilization Mechanism, and Training

The roll-out will begin with a joint TOT in Addis led by the FMOH, AHRI and LSHTM. Each study team will include medical and public health professionals experienced in clinical trials and NTD MDAs. The TOT will be an opportunity to review the training modules, assess the monitoring plan, and establish agreed upon courses of action for predictable eventualities.

Note that before the study begins, the FMOH and appropriate RHBs will work with the zonal and woreda-level offices where the study will take place to ensure full understanding and compliance with the study parameters. It is important to note that the communities in Kofele district will be excluded from the usual MDA training cascade which begins at the regional level and then descends down the zonal, woreda-level, and kebele-level tiers where Health Extension Worker (HEW) and Health Development Army (HDA) are the final training recipients. In place of this cascade, the FMOH/AHRI/LSHTM team will provide protocol specific training to the woreda health officers and the health extension workers. The training modules will include an overview of the national NTD training manual followed by an in-depth explanation of the study protocol and how it differs from the common MDA procedure. Training modules that will be modified from the usual NTD training will include:

- Given the possible pill burden of co-administration (up to nine pills for an adult), HEWs will be taught to allow beneficiaries a maximum swallowing regiment (two pills, swallow, two pills, swallow) in order to discourage rushed administration and any possibility of choking. HEWs will be taught to encourage patients to take one pill at a time if they prefer.
- The study team will train the HEWs and Woreda Health Officers on the protocol for reporting to the local medical team that will be stationed nearby during the study.
- The FMOH and the study team will create a mobilization plan with the HEWs that will rely on the Health Development Army and local leaders to create awareness of the study which in turn will help ensure participation. This will enable the project to get a collective informed consent for the activity by the community.

After getting the collective informed consent of the community, the HEWs will update the MDA register with the population and household information within the community as per the usual pre-MDA registration activities. While going house to house, the HEWs will read the study consent form to all members of the household and gain their verbal permission to participate in

the study. For those participants that refuse consent, they will be allowed to receive MDA treatment via the usual MDA schedule of IVM/ALB dose followed by azithromycin dose two weeks later.

8.3 Co-Administration Team Allocation

Once the training and social mobilization is complete, the directly observed MDA by co-administration of azithromycin, ivermectin and albendazole will be conducted through the regular channels of distribution via the community health structure. Within each of the two study arms, four teams will operate simultaneously within four separate gotes. At the actual distribution site, each team will address 300 people (roughly 60 households) per day. Assuming six hours (360 minutes) are allotted to each day for this co-administration exercise, each beneficiary would be allocated roughly 1.2 minutes to receive their treatment. In a normal MDA, this ratio would be easy to accommodate. However, given the need to complete the participant recruitment form (Appendix 2) for each beneficiary and to directly observe the administration of up to 9 pills (see the swallowing regimen referenced above), each team will form two, distinct distribution lines at the MDA site targeting 150 people each, resulting in a ratio of 2.4 minutes per beneficiary. The study will also only target 300 beneficiaries (60 HH) a day per team to facilitate the thorough follow-up of all 60 HHs the following day.

8.4 Recording of any AE/SAEs

The study defines AEs and SAEs per the national guidelines which are also found in the Serious Adverse Events handbook created by RTI ENVISION and the Task Force for Global Health[36]:

"**Adverse event from MDA (AE-F-MDA)** is a medical event that takes place in an MDA program, causes concern in the medical and wider community and is believed to be caused by the drug(s) used. It can be caused by either administration of the drug or by a coincidental event that by chance happened after drug administration. Most AEs-f-MDA (adverse events from MDA) are self-limiting and treatable using simple remedies and are not usually required to be reported to national or international regulatory authorities.

Serious Adverse Event (SAE) is a regulatory term describing any untoward medical occurrence with any of the following characteristics:

- Results in death;
- Requires in-patient hospitalization;
- Results in persistent or significant disability;
- Is life-threatening; or
- Results in a congenital anomaly/birth defect."

All adverse events will be recorded and followed up by the nurses on the team. The reporting of adverse events will be systematic and comply with national AE/SAE reporting guidelines to avoid any bias related to the wording of the question(s).

While the difference between "adverse events" and "serious adverse events" have been described above, there is also a level of severity which the study will also monitor: "The term "severe" is often used to describe the intensity (severity) of a medical event, as in the grading "mild", "moderate", and "severe"; thus a severe action need not be serious."[37] Reported adverse events will be classified according to their intensity (none, mild, moderate, and severe).

- Mild: easily tolerated, does not interfere with daily activity,

- Moderate: uncomfortable enough to interfere with daily activity,
- Severe: precludes daily activity (Given that a "severe" adverse event includes inability to complete daily activities, it will be classified as a possible SAE for the purposes of this study.)

The evolution of older complaints will be classified as: exacerbated, ongoing, and improved or resolved/none.

8.5 Analysis Plan of Reported AEs/SAEs

The study lead will conduct real time analysis of the data including rates and confidence intervals using the ODK system. (See **Section 8.9**) Adverse events will be coded per the WHO Adverse Reaction Terminology (WHO-ART) and Medical Dictionary for Regulatory Activities (MedDRA). They also be defined according to level of severity (mild, moderate, severe) as defined in Section 8.4. The study will create an independent Data Safety Monitoring Board (DSMB) composed of members of the FMOH as well as national and international medical experts. (See **Appendix 12: Charter of Data and Safety Monitoring Board**) As a safety measure, the study lead will convene an immediate teleconference with the medical doctor participating in the study and the DSMB given the following criteria*:*

- IF the rate of adverse events ranked as mild/moderate by participants differs significantly in the trial group as compared to the control group during the first two "Follow-up Days" **(Day 2, Day 4)** as well during subsequent review of the data at **Day 15**, and at the conclusion of the study on **Day 31** (to determine if the remainder of the woreda can safely be treated via triple drug therapy).

- IF an SAE occurs, the study lead and the medical doctor will conduct an immediate analysis and convene a call with the DSMB members. The DSMB members will advise the medical doctor in making an initial decision about attribution and whether a formal DSMB meeting is required. (Note: Given that a "severe" adverse event includes inability to complete daily activities, it will be classified as a possible SAE for the purposes of this study.)

- At conclusion of the study on Day 31 (to determine if the remainder of the woreda can safely be treated via triple drug therapy).

The causal relationship of serious adverse events observed with the triple therapy will be assessed as: *Unrelated, Unlikely, Likely*. The study will utilize information collected by the clinical nurses using the "Conduct an AE Investigation" steps detailed in the Serious Adverse Event Handbook. It is important to note that the study will not follow-up "minor" or "moderate" adverse events up with the full investigative checklist prescribed by the handbook but will reserve this for "severe" adverse events and SAEs. The PI, supervisors, and independent monitors will meet to review these determinations. If the supervisory group questions the results of the clinical nurses's investigations, it will perform its own investigation of the report as an addendum to the original report. Incidences will be analysed based on preferred terms and body system levels. "Severe" adverse events and SAEs occurring during exposures will be analysed based on 3 levels of selection per pre-existence and causality:

- Treatment emergent signs and symptoms (TESS), i.e., all AE during exposure to treatment or that were not pre-existing
- TESS without causality 'unlikely'
- TESS with causality 'likely'

8.6 Co-administration Team Composition

Both the trial and the control arm will be allocated 4 distribution teams. Each distribution team will be comprised of 8 people. This will result in 32 people on each arm with both arms together equalling 64 people. In addition, the study will assign an independent monitor to each arm of the study who will travel among the four teams and ensure that the study is implemented per protocol and with no bias.

8.6.1 Team breakdown:

2 clinical nurses

2 HEWs (or 1 HEW and 1 health officer)

3 HDA

1 supervisor

TOTAL: 8 members of on each team x 8 teams= 64 total team members

8.6.2 Team Description

2 clinical nurses: Before the drugs are given, the nurses (one for each line) will confirm that the beneficiaries have given assent and that there is a corresponding signature/mark for the community leader. The nurses will fill out the register questionnaire detailed in the appendix that establishes whether or not any previous medical conditions exist. Once the MDA portion is complete, the nurses will remain at the distribution site for the night in case any of the beneficiaries need to self-report adverse effects. The next morning, the nurses, with the aid of the HDA, will conduct house-to-house follow up visits according to the schedule detailed in the *Co-administration Schedule* section.

2 Health Extension Workers: HEWs (one for each line) will be responsible for actually co-administering the drugs to consenting/assenting beneficiaries. The HEW will assign the dosage according to different dose poles for IVM and azithromycin as well as the single dose of Albendazole. The HEW will be responsible for organizing the MDA schedule within the cohort as explained in Figure 1. The HEW will also be responsible for providing access for any reported AE/SAEs through passive follow-up should any community members have any complaints throughout the one month period.

3 HDA: Three members of the Health Development Army (one for each line and one to give line assignments to beneficiaries) will have the responsibility of assisting the HEW with community mobilization and crowd control once the co-administered MDA actually begins. These HDA members will also acts as guides to help the clinical nurses go house to house during the follow up day. The HDA will stay within each gote as this is their station. They will be within the community they are assigned for the rest of the study to provide passive surveillance.

1 supervisor: The study will assign one supervisor from the RHB, zonal office, or woreda health office to each team to ensure that the protocol is followed. The supervisors will report to the study managers who will travel between all the teams and provide general oversight and technical guidance.

8.6.3 External monitoring

The study will also invite independent study monitors to ensure that the trials is conducted in an un-biased manner. This will help encourage adoption of the triple drug therapy by the WHO assuming non-inferiority is established. The study team will invite these independent monitors from both academic institutions within Ethiopia and abroad. At a minimum, the study will assign one independent monitor to each arm of the trial.

8.6.4 Audits and Inspections

The study may be subject audit by the London School of Hygiene & Tropical Medicine under their remit as sponsor, the Study Coordination Centre and other regulatory bodies to ensure adherence to GCP.

8.7 Co-administration Team Composition

The co-administration study will place additional safety measures in addition to the National SAE/AE reporting protocol. As mentioned in the training section, all health extension workers and Health development armies within the targeted kebeles will be provided with a specially adapted training before the MDA addressing how to conduct the directly observed co-administration and how to administer the study questionnaire. Two clinical nurses for each of the four teams working in a cohort will supervise the co-administration and conduct the next-day follow up for all the beneficiaries that received triple drug administration. The study will also place a study physician at the study site for the duration of the 14-day co-administration exercise. The nurses will conduct active house to house surveillance starting the morning after the study and will regularly communicate with the assigned study physician stationed within the kebeles. A nearby referral centre (district/regional hospital) will be informed and on stand-by should any reactions occur. The study team will have three dedicated four-wheel drive cars to allow supervisors and study physician to travel between the targeted gotes and evaluate the work of the teams and for use in case evacuation to the health facility might become necessary.

8.8 Co-Administration Schedule

The study will have two cohorts control arm, which will receive the usual MDA, and study arm which will receive the triple co-administration. Each arm will have approximately 6,800 people, 42 Gotes. Primarily, the clustering will be made by geography where 42 adjacent Gotes will be in one and the other 42, in another. Which cluster will be the control or study arm will be decided by simple lottery method.

After decision on the study arms, the study team will conduct the training and the actual co-administration for a 17-day period (three days for training and 14 days for actual co-administration and surveillance). In total, the entire study will take 45 days to complete.

On Day 1, after the three-day co-administration training module described above**,** 60 households (HH) or roughly 300 people will be targeted for MDA subjects included in the study will receive concomitantly azithromycin (up to 1 g or four 250 mg tablets), ivermectin (up to 12 mg or four 3 mg tablets) and albendazole (one 400 mg tablet per included subject irrespective of height and age). This will mean that the four teams in the trial arm and the four teams in the control arm will have treated 1200 people each (300 x 4) by the end of Day 1. Teams will remain at the location of the MDA for the entire day and night on standby in case there are any immediate complaints from the targeted beneficiaries. Note that, as described in the “Intensified medical access precautions” section, a medical doctor will remain centrally located between within the woreda throughout the 14 actual days of distribution in the unlikely occurrence of a reaction to the medication.

On Day 2, the clinical nurses on each team, led by the three HDA members, will travel to all 60 households within their team’s catchment area to confirm that all 300 persons that received drugs on *Day 1* are all healthy and to document any AEs/SAEs that may have occurred. This will also serve as opportunity for a person-to-person coverage survey for later analysis. At the end of the day, the four teams from each arm, together with the independent monitors, PI, supervisors, etc. will meet and discuss if there were any results that should cause concern. While the clinical nurses conduct the house-to-house visits, the HEWs on each team will move to the next group of 60 households targeted for the Day 3 MDA and mobilize that community.

On Day 3, each team will treat an additional 300 people (60HH) following the same regimen as was described on Day 1. By the end of Day 3, the four teams in the trial arm and the four teams in the control arm will have treated 1200 people each (300 x 4) Together with the people treated on Day 1, this will equal 2400 people out of the 16, 000 people targeted for treatment.

On Day 4, the clinical nurses on each team will once again travel to the 60 households of the 300 beneficiaries within their catchment area and follow the procedure described on Day 2. All 1,200 people treated on Day 3 will receive a house visit. The HEW will mobilize the community targeted for MDA on Day 5.

On Day 5, each team will treat an additional 60 households or roughly 300 people. Across the four teams in each arm, this will equal roughly 1,200 people each (4 teams x 300 people) and, when added together with the populations treated on Day 1 and Day 3, will equal 3,600 people (Day 1: 1,200 + Day 3: 1,200 + Day 5: 1,200= 3600 people).

On Day 6, the clinical nurses in all six teams will once again follow up with all people treated on Day 5. *Note on Day 6 and 7, the doctor as well as the PI and a woreda health officer will travel back to the communities treated on Days 1-5 to gather any passive AE reports from the HDA members originally involved in the distribution to that community.*

On Day 7, Day 9, Day 11, Day 13: same MDA distribution as described on Day 1. When combined with the 5,400 people from Days 1,3, and 5, this will result in a total of 12,600 people targeted for treatment. (5,400+ 100 +1800 +1800+1800= 12,600).

On Day 8, Day 10, Day 12, and Day 14 Same active house to house follow-up method described on Day 2, 4, and 6.

By the end of Day 14, the actual triple-drug co-administration will be completed. On Day 14 and 15, the medical doctor, PI and woreda health officer will once again travel to the communities treated between Day 7 and 14 to gather any reports from the passive

surveillance carried out by the HDA. The HEWs and clinical nurses will continue observation in Kofele for an additional 15 days per the following schedule:

On Days 15 and 16, the HEW and clinical nurses will remain stationed at a central location between the targeted gotes in case any of the beneficiaries has symptoms to report that might be linked to the co-administration. This will be a system of passive, self-reporting follow-up.

On Day 17, 18, the clinical nurses and HEW from each of the original four teams will perform one last house to house follow up of all the households that received the triple drug administration. (400 HH x 4 teams = 1600 HH) This will be the last active follow-up step.

Day 19-30, the HEWs will maintain the reporting form for another two weeks in the event of any additional complaints from the targeted beneficiaries. This will be completely passive and the HEW will only log complaints if they are reported by a beneficiary. On Day 30, all of the co-administration registers and forms will be collected for data analysis from the HEWs.

Note that in the “standard MDA” control arm, the process described above will be conducted twice. First, IVM/ALB MDA will take place for with 14 days of active surveillance then the Trachoma MDA will take place for 14 days of active surveillance. Passive surveillance will continue in the control arm for two weeks after that.

Once the 45th day of the study is reached in Oromia, the field implementation portion of the study will be complete. The DSMB will review the final data before making a recommendation on how the remainder of the population in the woreda should be treated. Should the DSMB and the FMOH agree, the remaining 4-5000 beneficiaries within Homesha will be targeted for co-administration. However, at this juncture, the RCT will be over. The study will move to strictly observational with the same enhanced supervision in place.

Table 3: Follow up Schedule for both the Trial and the Control Arms of the Study

<table>
<tr><th></th><th>Day 1</th><th>Day 2</th><th>Day 3</th><th>Day 4</th><th>Day 5</th><th>Day 6</th><th>Day 7</th><th>Day 8</th><th>Day 9</th><th>Day 10</th><th>Day 11</th><th>Day 12</th><th>Day 13</th><th>Day 14</th><th>Day 15</th><th>Day 16</th><th>Day 17</th><th>Day 18</th><th>Day 19-30</th><th>Day 31</th></tr>
<tr><td>Actual Distribution</td><td style="background:yellow"></td><td></td><td style="background:yellow"></td><td></td><td style="background:yellow"></td><td></td><td style="background:yellow"></td><td></td><td style="background:yellow"></td><td></td><td style="background:yellow"></td><td></td><td style="background:yellow"></td><td></td><td></td><td></td><td></td><td></td><td></td><td></td></tr>
<tr><td>Next Day Follow-up</td><td></td><td style="background:red"></td><td></td><td style="background:red"></td><td></td><td style="background:red"></td><td></td><td style="background:red"></td><td></td><td style="background:red"></td><td></td><td style="background:red"></td><td></td><td style="background:red"></td><td></td><td></td><td></td><td></td><td></td><td></td></tr>
<tr><td>One week follow up</td><td></td><td></td><td></td><td></td><td></td><td style="background:green"></td><td style="background:green"></td><td></td><td></td><td></td><td></td><td></td><td></td><td></td><td></td><td></td><td></td><td></td><td></td><td></td></tr>
<tr><td>14 Day Follow up</td><td></td><td></td><td></td><td></td><td></td><td></td><td></td><td></td><td></td><td></td><td></td><td></td><td></td><td style="background:green"></td><td style="background:green"></td><td></td><td></td><td></td><td></td><td></td></tr>
<tr><td>Passive Surveillance</td><td></td><td></td><td></td><td></td><td></td><td></td><td></td><td></td><td></td><td></td><td></td><td></td><td></td><td></td><td></td><td></td><td></td><td></td><td style="background:brown"></td><td></td></tr>
<tr><td>DSMB Review (or as needed via charter description)</td><td></td><td></td><td></td><td></td><td></td><td></td><td></td><td></td><td></td><td></td><td></td><td></td><td></td><td style="background:steelblue"></td><td style="background:steelblue"></td><td></td><td></td><td></td><td></td><td style="background:steelblue"></td></tr>
</table>

Figure 1: Randomization and MDA distribution Plan in Trial and Control arms

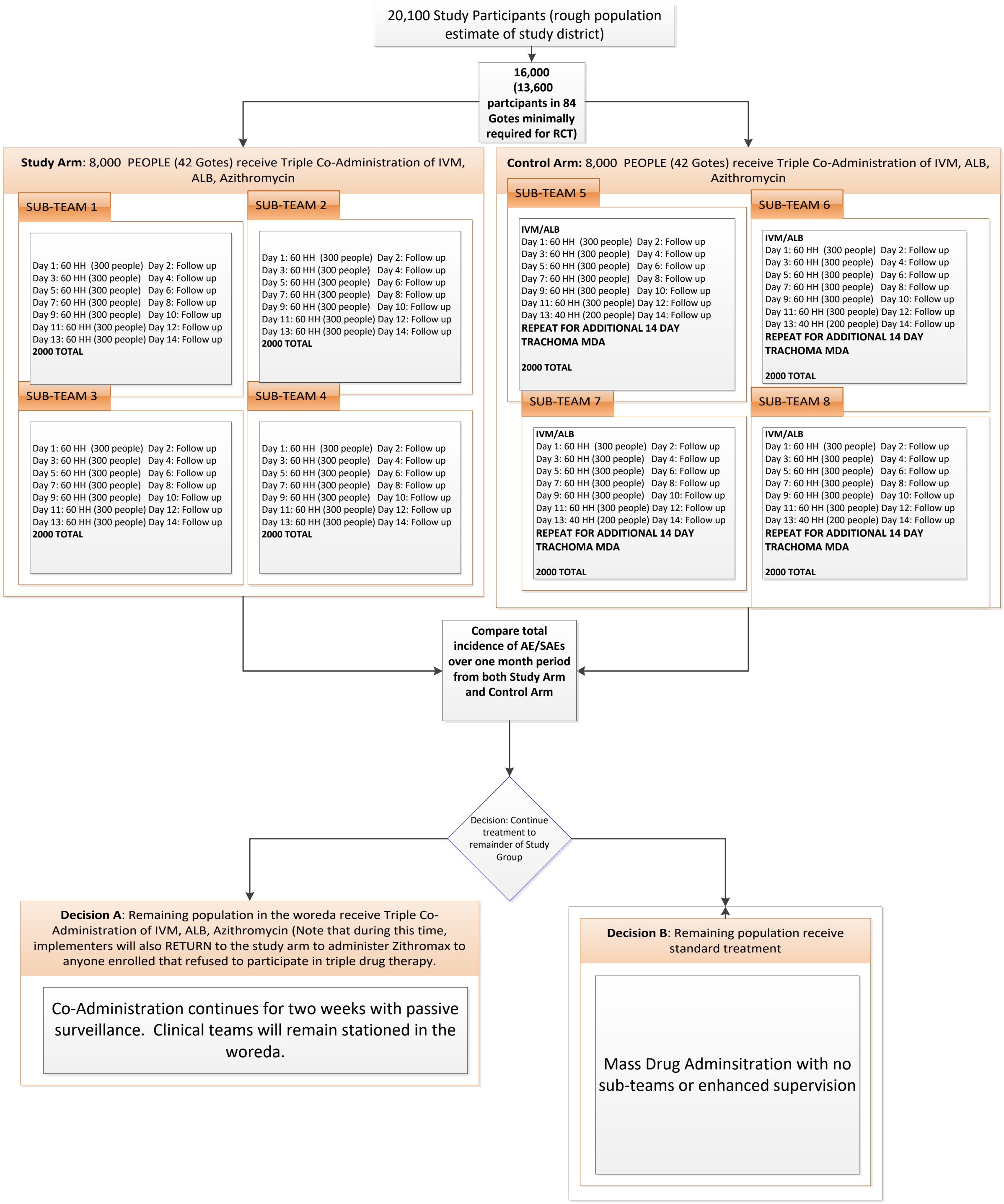

8.9 Data Collection and Statistical Analysis

Data about individual participant will be collected by the study team using structured questionnaires and coded, de-identified labels which will be held in strict confidence. All results will be presented in a way that does not allow individuals to be identified. No information concerning the study or the data will be released to any unauthorized third party. These questionnaires are presented in the appendix of this protocol. The study team will rely on electronic data capture and will convert these forms to the ODK electronic format at the London School of Hygiene and Tropical Medicine.

Table 4: Data Collection Summary for AE reporting

**Forms**	**Timeline**	**Person responsible for filling out the form**
Informed Consent	Pre-MDA Census days and Distribution Day	HEW/Clinical Nurses
Socio-demographic/health Information	Distribution Day	Clinical Nurses
Study drug registry	Distribution Day	Clinical Nurses
Contra-indication/AEs reporting to the Co-Administration team (HEW and clinical nurse)	Day 2, Day 4, Day 6, Day 8, Day 10, Day 12, Day 14, Day 17+18 follow up or at any time during passive surveillance	Clinical Nurses/HEW
Contra-indication/AEs reporting to the local health post (HEW)	Day 19-30	HEW

* *Study team will return to each health post at 1 month after the MDA to collect information on reported AE/SAEs*

Data will be entered in STATA. All variables will be summarized using descriptive statistics as is appropriate. The co-administration safety will be characterized as safe or unsafe.

Parameters to be determined are:

- Population of study kebeles: composition by age group and sex.
- Prevalence and incidence of adverse events by age group, sex, and kebele
- Severity of adverse events in each kebele
- Average age of participant experiencing adverse events

The null hypothesis is that the occurrence of adverse events will be significantly higher in the co-administration trial communities in comparison to the standard MDA control communities. The study will report the proportion of individuals in each arm experiencing an AE adjusting for

clustering. It will also report the proportion of AEs stratified by age group. The test statistic will be clustered logistic regression using the covariates of severity and treatment package.

It is important to note that we consider that if the number of adverse events in the co-administration arm was markedly higher than in separate administration arms, for example >5% absolute difference in AEs, than the increased number of adverse events would likely outweigh any logistic or cost benefits. However, a stricter non-inferiority margin of 1.5% was used as the basis for the sample size calculation and was selected for two reasons:

1) If the trial results in non-inferiority at this level the study believes this represents unequivocal evidence in favour of co-administation; i.e given the absence of a marked increase in adverse events, the logistic and financial advantages mean the rationale in favour of co-administration is clear.

2) To provide a more precise estimate of the difference in the proportion of adverse events in the control and intervention arms.

If the trial were not to meet this non-inferiority margin (i.e the upper limit of the 95% CI crossed our specified delta of 1.5%) but did not cross the threshold at which the increase in the number of AEs was clearly unacceptable (5% difference) then the study believes that plausibly co-administration might still be favourable even accepting that it did result in more adverse events were the logistic and financial benefits to be marked.

9 Ethical Clearance

For this study, LSHTM and the FMOH have joined together with the Armauer Hansen Research Institute (*AHRI- see Appendix 1 for full description of AHRI*). The study will first seek ethical clearance from LSHTM as per the requirements of both the donor (BMGF/ITI) and the PhD program. First and AHRI will then be responsible for gaining ethical clearance via the AHRI ethical review board. Once the ethical review has been approved, AHRI will approach then approach the Food, Medicine and Health Care Administration and Control Authority (FMHACA) review board which must approve any studies involving pharmaceuticals. Oral informed consent will be obtained from all adult study participants, and parents/guardians will provide informed consent for their children, while at the same time the children themselves will assent to participating.

10 Timetable for Study Completion

TASK	START	END
Phase 1: Study Preparation		
Waiting for ethical approval	9-Oct-21	18-Nov-21
Securing drug supply	9-Oct-21	18-Nov-21
Field site visit	1-Nov-21	7-Nov-21
Recruiting from local health care	1-Nov-21	7-Nov-21
Training	8-Nov-21	14-Nov-21
Transporting logistics	8-Nov-21	14-Nov-21
Mobilizing community for Day 1 of study	17-Nov-21	17-Nov-21
Phase 2: Study Period		
Conduct Co-Administration study in Kofele	18-Nov-21	31-Dec-21
Phase 3: Analysis and Publication		
Analysis of Data	1-Jan-22	30-Jan-22
Write up/Dissemination Meeting	15-Jan-22	31-Jan-22

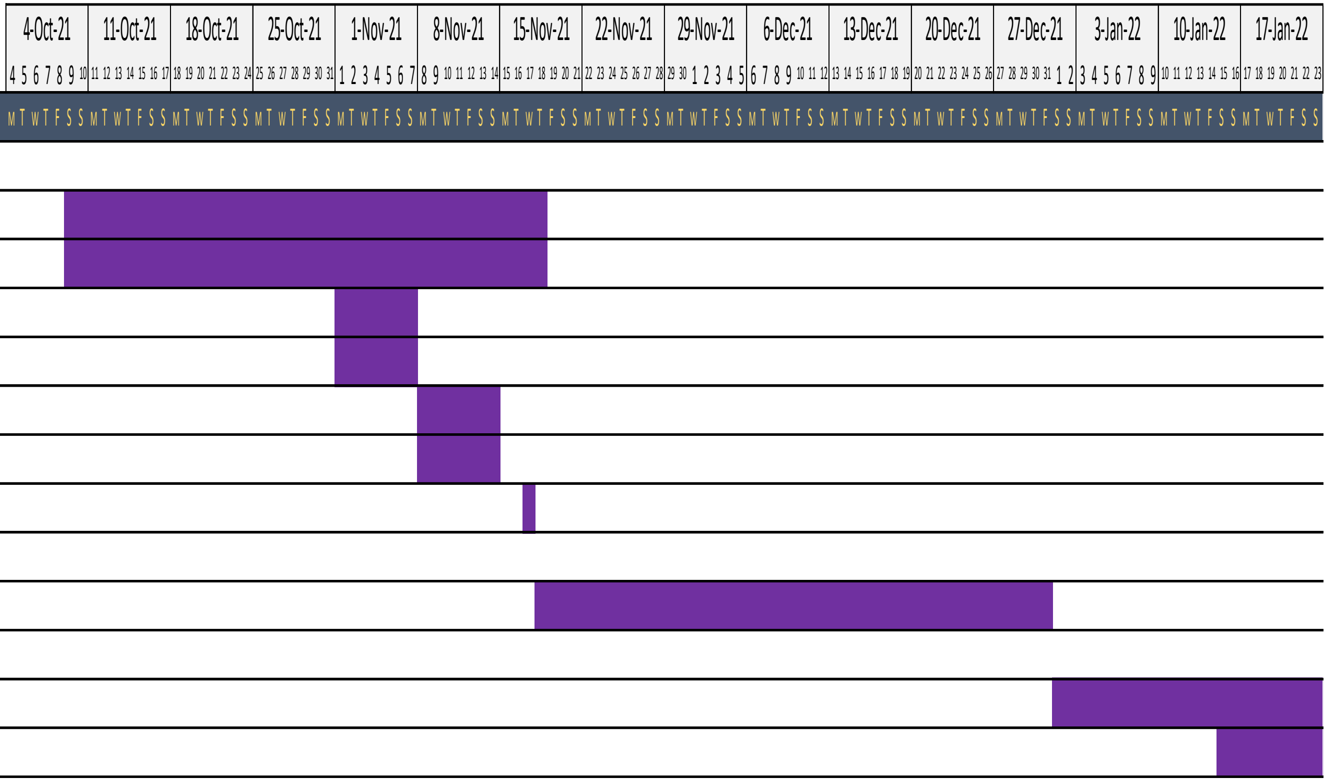

The Primary outcome is

- The safety as can be measured by incidence of AEs/SAEs over one month following the co-administered MDA in comparison to a control group receiving the standard MDA regimen.

Secondary Outcomes are:

- Understanding the perceptions of the beneficiaries receiving the co-administration of azythromycin, ivermectin, and albendazole especially surrounding pill burden and the change in MDA distribution schedule.
- Understanding the perceptions of the co-administration strategy by the local health workforce as a means of PC-NTD drug delivery.
- A cost analysis comparing existing MDA unit costs from woredas within the region already on a standard MDA schedule with that of the co-administered woredas.

11 Secondary Outcomes

I.) Better understand the perceptions of the beneficiaries/recipient population of co-administration of azithromycin, ivermectin, and albendazole especially surrounding pill burden and the change in MDA distribution schedule.

Background: Currently, mass drug administration takes place through three distinct phases depending on the number of NTDs for which a woreda is endemic. Assuming a community is endemic for trachoma, LF, and OV, the first MDA round that takes place addresses OV an LF through a height-dependent dosage of up to four pills of ivermectin and, regardless of height or age, one pill of albendzole. The second round of MDA takes place two weeks later for the administration of azithromycin involving a height-dependent dosage of up to four pills for azithromycin (with TEO administered to children under a height of 48 cm). According to the Ethiopian national strategy to eliminate onchocerciasis, almost all of the OV endemic woredas in Ethiopia have been moved to a twice-a-year treatment strategy. Notably, though STH and SCH are usually treated through school-based MDA, communities that are co-endemic for STH with prevalence over 50% and IVM will receive a second round of albendazole (or mebendazole) together with the OV second round of ivermectin.

The Challenge: While cost, supply chain, and the work load of the local health work force are important considerations (and further explored through the other secondary outcomes of the study in this section), the most important aspect to consider is that of the beneficiary. Depending on the dosage, determined by a beneficiary's height for ivermectin and azithromycin, an adult of certain height will find him or herself taking up to nine pills (four for ivermectin, four for azithromycin, and one for albendazole). While the swallowing regiment (a maximum of two pills at a time or one at a time according to the beneficiaries' comfort level) is meant to control for the possibility of choking during MDA, taking up to nine pills at one time can create both physical and mental duress. The level of duress, if any, must be ascertained and considered in conjunction with the physiological assessment of side effects and the operational benefits before any adoption of the co-administration strategy can take place by a national program.

Study Goal: Triple drug administration may present an opportunity to significantly lessen the time investment required for beneficiaries to participate in all of the necessary mass drug administrations, time that could otherwise be used for farming, time at the market, etc. Lessening the time a beneficiary needs to invest to receive drug administration may subsequently improve MDA coverage.

This sub-study will seek to discover, through a series of in-depth interviews and focus groups within the trial arm the benefits and disadvantages of the co-administration strategy.

Study area: The study will take place within the same gotes selected for the trial arm

Study Sample: The target participants will be selected among the beneficiaries living within the gotes where the co-Administration of azithromycin, ivermectin and albendazole took place. In order to ensure that experience is not influenced by possible logistic or programmatic challenges unique to a given area, selection of participants will be randomized using the RANDNUM function from a total list of the 8000 participants derived from the pre-MDA census. This process will be continued (excluding any beneficiaries already chosen) until a total of 100 interviewees have been selected.

Patient Interview Structure: Patients will be interviewed within the community by an Amharic-speaking interviewer as well as an interpreter fluent in the mother tongue of the area. It is important to note that Affan Oromo is the *lingua franca* for Oromia. The patient interviews will take place within the interviewee's home or in a private area surrounding the distribution point, and in an environment which is secluded from members of the surrounding community in order to discourage peer and community influence on the interviewee. The interviews will take place either immediately after the beneficiary receives his/her co-administered dose or during the post-MDA active follow-up that will take place the following day. Written consent will be obtained from all participants using the form in **Appendix 9**. While the interview structure will seek to move beyond yes/no questions and foster real discussion, basic topics will include: 1.) A description of the recipient's "average day" in terms of work, child-rearing, etc. 2.) A description of what the recipient believes are the biggest health issues within the community, 3.) The recipient's thoughts on the role of the HEW, 4.) Perceptions surrounding the "usual" MDA distribution and how it affects the "average day" of the recipeient, 5.) Perceptions of "usual" MDA versus the co-administration, 5.) Perceptions of pill burden, both for the recipient and the recipient's family. Interviewers will be trained in the reduction of biases when conducting these interviews.

Study Analysis: All interviews will be recorded and transcribed. I will sort through all of the transcribed interview data and flag statements from participants that are significant/insightful/often repeated. Using qualitative software, either MAXQDA or NVIVO, I will create both pre-defined codes based off of identified themes (via both published literature and practical experience in Ethiopia) and from the data up. Given that Ethiopia has an established history of MDA but also given that co-administration has never taken place before, I anticipate that certain codes surrounding MDA can be previously identified while emerging codes from the data up exercise will be important. I will also create categories for "descriptive" codes relating to the participants themselves (age, gender, etc.) and "thematic" codes relating to what was actually said by the participants. I will assign the previously identified "significant statements" of the participants to the codes, likely resulting in one statement having several different codes attached. Once coding is completed, I will explore any patterns in responses, linkages between the thematic and descriptive codes, and often repeated statements which may require follow up to avoid generalizations. It is important that the study and the coding is completed while still in the communities as I intend to conduct member checking of the results with the original participants to increase the validity of the studies.

II.) Better understand the perceptions of the co-administration strategy by the local health workforce as a means of PC-NTD drug delivery

Background: The FMOH and RHBs currently carry out many health initiatives at three levels: Primary Health Care Units (PHCUs), the Health Extension package, and the Health Development Army (HDA). PHCUs are woreda-level medical clinics. On average, there are five PHCUs per woreda. The Health Extension Program, which was created to address medical intervention needs at the community level, consists of an integrated set of 16 health packages, including NTD intervention through MDA. The

FMOH has trained and deployed approximately 38,000 health extension workers (HEWs) across the country to implement these health packages. They are government-salaried, community-based health workers. The HDA is a community-level cadre composed of six women health volunteers per community. Each member of an HDA is assigned five households. The HEWs lead groups of HDA members to form health development teams. Overall, there is an average of 30 development teams in each kebele. Given that HEWs are salaried positions, they are hired by the woreda health office through a competitive interview process while HDA volunteers are recognized as "community influencers" and are selected by community leaders. Both HEWs and the HDA are predominantly female.

In terms of NTD interventions, use of the HEWs and members of the HDA is very effective. HEWs conduct all of the MDA registrations and supervision while the HDA assists with mobilization and directly observed treatment. Although HDAs can administer albendazole and ivermectin, they cannot administer azithromycin because it is an antibiotic. This task is left to the HEWs. Mebendazole and praziquantel are distributed by teachers through school-based distribution except in woredas with high-risk groups or a prevalence over 50%, in which case the HEWs lead community-wide distributions. The FMOH adopted a campaign-style MDA in 2013 using the HEWs, HDAs, and teachers for all NTDs. The move away from "rolling" MDAs, which was supported by the Community Directed Treatment with Ivermectin (CDTI) strategy, has been very successful in reducing the average time for MDAs from 1 month to 5 days to cover the same area.

Table 3. Official MDA calendar from the FMOH

Program	Round 1	Round 2	Remarks/Justification
**Trachoma:** Community-based distribution performed by HEWs with mobilization assistance from the HADHDA	October/November and March/April		This allows adequate time interval between trachoma and other preventive chemotherapy (PC) MDA.
**SCH and STH:** School-based deworming conducted by teachers with supervision from HEWs	October (Round 1 for STH in twice-per-year woredas)	First week of April (Round 2 for STH in twice-per-year areas)	This schedule ensures that all school-aged children (SAC) are covered at the beginning of the school year, thereby improving learning throughout the academic year.
**OV and LF:** Community-based distribution performed by the HDA with supervision from HEWs	October (for all OV- and LF-endemic areas)	First week of April for OV Round 2 only	LF-endemic woredas included in Round 1 MDA will have the ancillary benefit of addressing STH.<br>Round 2 OV MDA will be undertaken without ALB.

The Challenge: With more than 80 million people at risk for at least one NTD, the investment of time and human resources to conduct all of the necessary campaigns is massive. Every region currently follows its own MDA schedules determined by drug availability and the schedules of other community health initiatives. With MDA often required twice a year for OV, once a year for LF, possibly twice a year for STH/SCH depending on prevalence, and once a year for trachoma, NTD interventions are quickly becoming one of the greatest demands on the community health infrastructure. HEWs, the backbones of the MDA mechanism, may be called out of their health post to attend woreda-level NTD trainings and post-MDA reviews four or five times a year within a single woreda, meaning that the community is left without one of their healthcare providers.

Study Goal: Triple drug administration may present an opportunity to significantly lessen the work burden demanded of HEWs by NTD work in that it combines the two separate MDAs of OV/LF and Trachoma, usually provided two weeks apart, into one. This includes the reduced training time and supervisory requirement of the combined MDA schedule. However, it is also possible that the administration of so many drugs at the need to more stringently observe the various exclusionary criteria of all three drugs will present a greater burden to HEWs. For this reason, the study team composed of the FMOH, LSHTM and AHRI, will conduct focus group discussions with HEWs that have experience both with the standard MDA schedule and the co-administration study criteria in order to ascertain how HEWs view the co-administration methodology from a quality and quantity of work standpoint.

Study area: The study will take place within the same gotes targeted for co-adminsitration

Study Sample: Participants will be members of the Health Extension Work force employed within the kebeles where the co-administration of azithromycin, ivermectin and albendazole took place. The study will conduct a case studytargeting 12 individual interviews during the case study component followed by two FGD composed of six people each drawn from among the HEWs assigned to the cohort area. In order to ensure that experience is not solely based on logistic or programmatic challenges within a given area, selection of HEWs will be randomized using the RANDNUM function from a list of all of the HEWs participating in the study. This process will be continued (excluding any HEWs already chosen) until 24 (12 for the case study and 12 for the FGDs) HEWs have been selected. Further selection criteria will include: 1.) An HEWs availability to participate in the study at a given time (given the requirement that two HEWs are usually placed within a health post and one must always be "on call" and thereby unavailable to participate). 2.) At least one year's prior experience within the area as an HEW which includes direct participation in all MDA rounds (for OV/LF, STH, Trachoma and LF) occurring within the catchment area. These criteria will be confirmed by the employment register at the woreda health office and/or by calling the selected HEW. Any HEWs that are excluded because they do not meet the criteria will be replaced through the aforementioned selection process. Written consent will be obtained from all participants before the study using the form in **Appendix 9**.

Study Structure

HEW Case Study Structure

HEWs that participated in the co-admin study but were not among those randomly selected for the focus groups discussion will be selected for a case study exercise. The project will interview these "case study" HEWs before, during and after the MDA via semi-structured interviews. Before the interviews begin, each participant will fill out a basic demography and work experience form. Written consent will also be collected from each participant. Every session will be recorded in its entirety. These interviews will encourage the HEWs to reflect on 1.) Why they became HEWs, 2.) The type of relationship they have with political leaders and community influencers (local healers, politicians, teachers, etc.), 3.) how they believe HEWs are perceived by the community, 4.) what the major health issues facing the community and where, if at all, NTDs rank within those issues. 5.) HEW's average daily and monthly work-related responsibilities 6.) How do HEWs view MDA in terms of time investment and community benefit. The results of the first interview, held before co-administration takes place, will inform the structure for the "during co-administration" interviews and the "post co-administration interviews" as well as the questions for the focus group discussions detailed

below. The "during co-administration" and "post co-administration" questions will include inquiries into how HEWs feel about the co-administration strategy.

HEW Focus Group Structure

As with the HEW case study, before the focus group begins, each participant will fill out a basic demography and work experience form. Written consent will also be collected from each participant. Every session will be recorded in its entirety. Each focus group will be semi-structured and led by an experienced moderator and dictated by an additional note taker. The study team will work with LSHTM and the FMOH to create a logical flow model that will carry the moderator from one topic to the next without hindering free flow of thought from the focus group. The topics will move from a more general nature to specificity surrounding the co-administration and will be heavily influenced and edited based on the experience of the "pre-MDA" case study interviews. Discussions will take place within a community setting, most likely at a health post or health center that is centrally located between the six selected HEWs and their catchment areas.

Study Analysis: Analysis of the FGD and Case Study data will follow the same process described under Secondary Outcome 1.

III.) A cost analysis comparing existing MDA unit costs from woredas within the region already on a standard MDA schedule to a projection of what those cost would be under the triple drug strategy.

Background

As noted in *Table 2*, there are several woredas with co-endemic permutations of onchocerciasis, lymphatic filariasis, soil transmitted helminths, and trachoma in Ethiopia which would make strong candidates for the implementation of the triple drug co-administration strategy. In addition to the perceptions of the community and the health work force (detailed above), it is important to understand what programmatic benefits a national program could anticipate by implementing the co-administration strategy. From a programmatic perspective, perhaps the most important of these benefits are the possible fiscal savings that might result from aligning the costs of several stand-alone MDAs, including logistics, supervision, training, etc., into one combined activity.

Study Design

In order to assess possible savings surrounding the co-administration strategy, the study will draw from several budget sources:

Tool for Integrated Planning and Costing (TIPAC): RTI International, through support from the United States Agency for International Development (USAID), created the TIPAC as a tool that allows national programs to more accurately budget for NTD interventions and quantify the costs surrounding any programmatic gaps.[4] Among other uses, the tool uses cost categories and sub-categories to build a comprehensive budget for projected activities. In order to populate these cost-categories, a national program must conduct preliminary costing exercise to derive average unit costs. In 2013, RTI International conducted a workshop in support of the FMOH of Ethiopia to fully populate its own national TIPAC. RTI also provides a seconded data manager to the FMOH to update the TIPAC regularly both with revised unit costs and budget information on new activities (e.g. - lymphedema management unit costs were added

[4] http://ntdenvision.org/sites/default/files/docs/tipac_3.0_en_letter.pdf

to the tool in September 2016). For the purposes of the study, the TIPAC unit costs for disease-specific MDAs which include such categories as transport, supplies, supervision, and training will serve as one base of comparison to the co-administration costs.

Implementing partner costs: While the TIPAC is useful for national-level planning, it does depend upon unit cost averages. Since these averages are created with costing information from around the country, there can be varying applicability when attempting to assign the TIPAC unit costs to a specific zone or region. For this reason, the study will also draw upon the costing information of the different implementing partners that support NTD MDA within the woredas and zones targeted for the co-administration study.

The study will use the national MDA unit costs and the implementing partner unit costs as two separate means of comparison with the co-administration costs. Given that the triple drug co-administration protocol will require extensive additional supervisory mechanisms and logistic support, the unit costs derived from the study will not reflect what an actual co-administration might cost once safety has been established and the practice is integrated into the national health system. Therefore, the study will create a theoretical unit cost frame work for the co-administration exercise making certain assumptions about what triple drug co-administration will require once it is a standard practice. Once the trial is complete, it may turn out that certain additional components, such as extra staff to ensure the “swallowing regiment” is properly implemented, etc., will require an increase in unit costs from that of a standard MDA.

SUPPLEMENTARY APPENDICES

Appendix 1: Partner Organizations participating in the study (largely copied from Institution Websites):

Federal Ministry of Health NTD case team

Background: The Federal Ministry of Health NTD case team leads the nationwide campaign to combat NTDs. The case team conducts evidence-based planning and implementation of strategies to address lymphatic filariasis, onchocerciasis, trachoma, soil transmitted helminths, schistosomiasis, visceral and cutaneous leishmaniasis, and podoconiosis throughout the country. The case team was created by the Hon. Minister of Health in 2013 following the National NTD symposium and launch of the National NTD Master plan.

Study Role: The Federal Ministry of Health requested this study as one of its priority research questions. The FMOH will be responsible for garnering the full support of this study from the National Trachoma Task Force (NTTF) and the Regional Health Bureaus (RHBs) in the region where this study will take place. The FMOH will also be directly involved in every phase of implementation of this study.

Armauer Hansen Research Institute (AHRI): (http://www.ahri.gov.et)

Background: AHRI is a biomedical research Institute in Ethiopia specializing in biomedical and clinical investigations, including clinical trials. In addition to the clinical trials group, AHRI has specialized research groups focusing on: non-communicable mycobacterial diseases, bacterial and viral diseases, malaria, neglected tropical diseases, and biotechnology/bioinformatics. Each research group includes rigorous training and research capacity building component. More than 670 papers in peer reviewed journals have been published from AHRI during its 45 years of existence. In addition, it has also produced a substantial number of theses and dissertations from international and Ethiopian scholars in biomedical research. The AHRI NTD team has done nationwide risk mapping and validation studies and accumulated experience in working with communities, including with those in remote areas.

Study Role: AHRI will co-implement this study with the LSHTM. First and foremost, AHRI will be responsible for co-designing the protocol, gaining ethical clearance and arranging for the logistics. AHRI will also provide day to day management of the medical response and survey teams on the ground. As this study will also serve as a capacity building initiative, LSHTM and AHRI will work together to find opportunities within the study for AHRI's up and coming researchers.

London School of Hygiene & Tropical Medicine – United Kingdom

(http://www.lshtm.ac.uk/aboutus/introducing/mission/index.htm)

Background: The London School of Hygiene & Tropical Medicine is a world-leading centre for research and postgraduate education in public and global health. The London School is a WHO Collaborating Centre for Trachoma and played a major role in the Global Trachoma

mapping project. They have recently led a study on co-administration of Azithromycin and ivermectin in the Solomon Islands.

Study Role: The London School of Hygiene and Tropical Medicine will co-implement this project with AHRI and advise on the study design and implementation of the project.

International Trachoma Initiative – United States of America (http://trachoma.org/about-iti)

Background: The International Trachoma Initiative (ITI) was founded in 1998 in response to the World Health Organization's (WHO) call to eliminate blinding trachoma by 2020 (GET2020). ITI's founding partners, Pfizer and the Edna McConnell Clark Foundation, saw the need for an international nongovernmental organization dedicated solely to the elimination of blinding trachoma. To achieve that goal, ITI collaborates with governmental and nongovernmental agencies at the local, national and international levels to implement the WHO-recommended **SAFE** strategy for trachoma control (**S**urgery; **A**ntibiotics—using donated Zithromax®; **F**acial cleanliness; and **E**nvironmental improvement).

Study Role: ITI will provide the funding for all aspects of this study and advice on the study design and implementation.

NOTE: All of the forms listed below have been adapted together with AHRI based on previous clinical trial reporting forms. It is the desire of the FMOH and study leads to combine these questionnaires into one comprehensive register which, if approved by the in-country ethical review board, will be converted for electronic data capture during the study.

Appendix 2: Case recording forms

Form 1: Recruitment form

Study Title: Safety of the co-administration of azithromycin, albendazole and ivermectin versus standard treatment regimens during mass drug administration (MDA) in Ethiopia: a cluster-randomized trial

Name of investigator: ……………………… Name of supervisor: ……………………

I. Scociodemographic Data

1.1. Community ID: |__ |__||___||__ |__||___|

1.2. Participant ID |__ |__||___||__ |__||___||__ |__||___|

1.3. Date of birth |_DD| MM | YY |

1.4. Approximate age if date of birth is unknown:|__ |__|.|___| years

1.5. *Height* :|__ |__||___| .|___||___| Cms

1.6. Sex: 1. Male 2. Female 99. Unknown

1.4. Household code…………..

1.5. Gott: ……………………………………………… 1.6. Kebele………………………………………………

1.7. District…………………………………………………1.8.

Region…………………………………………………………

1.9. Consent Card Number: |____ |____ |____ |____ |

1.10. Survey Sheet Number: |___ |____ |____ |____ |

II. Interview prior to treatment: signs/symptoms or complaint

2.1. Signs and symptoms at presentation: **Tick any as is appropriate**:

	**Events prior to treatment**	**existing prior to treatment**	
	**Symptoms/Signs**	**1. Yes**	**2. No**
**2.1.1.**	Fever		
**2.1.2.**	Itching/ground itch		
**2.1.3.**	Headaches		
**2.1.4**	Dizziness		
**2.1.5**	Tiredness		
**2.1.6.**	Deafness		
**2.1.7.**	Jaundice		
**2.1.8.**	Weakness		

2.1.9. Fatigue

2.1.10. Nausea

2.1.11. Vomiting

2.1.12. Diarrhoea

2.1.13. Abdominal pain

2.1.14. Flatulence/dyspepsia

2.1.15 Constipation

2.1.16. Joint/muscular pain

2.1.17. Swelling of (upper/lower) limbs

2.1.18. Swelling of eyelids/abnormal feeling in the eyes

2.1.19. Rash/plaque

2.1.20. Scrotal reaction

2.1.21. Skin nodules

2.1.22. Worm expulsion

2.1.23. Haematuria

2.1.24 Lymphoedema

2.1.25 Elephantiasis

2.1.26. Hydrocele

2.1.27. Palpitation/tachycardia

2.1.28. Orthostatic hypotension

2.1.29 Others

2.1.30. **If other specify……………………. ....**
... .
....

2.2. ***After completing all Questions under 2.1, the investigator should answer the questions that follows: NB:-the answer will be yes if one complaint is ticked in the above list***

2.2.1. Did the participant have complaints prior to the treatment? 1. Yes 2. No

2.2.1.1. Indicate the **Main complaint**……………………………………………………

2.2.2. How would you rate this complaint? **1 = minor; 2 = moderate 3=major**

2.2.3. Is volunteer eligible for the study? 1 Yes 2 No 99 Unknown

2.2.4. Is informed consent/assent obtained? 1 Yes 2 No 99 unknown

Thank her/him for volunteering and accompany him/her to the treatment station with his/her file

Appendix 3: Treatment form

2.1 **Scociodemographic data**

2.2 Community ID: |__ |__||___||__ |__||___|

2.3 Participant ID |__ |__||___||__ |__||___||__ |__||___|

2.1.3. Date of birth |_ DD| MM | YY |

2.1.4. Approximate age if date of birth is unknown:|__ |__|.|___| years

2.1.5. Sex: 1. Male 2. Female 99. Unknown

2.1.6. Household code…………..

2.1.7. Gott: ……………………………………………….. 2.1.8. Kebele……………………………………………….

2.1.9. District... 2.1.10. Region...

2.1.11. Height: |___|____|____|_____| CM

2.1.12. Weight: |___|____| kg

2.1.13. Number of tablets taken:

2.1.14. IVM: |___|

2.1.15. ZI: |___|

2.1.16. ALB: |___|

2.1.17. Date of DOT co-administration|_ DD| MM | YY |

2.1.18. Time of DOT co-administration |___|____|.|____|_____|hrs

2.2. Implementing physician/health professional directed information

2.2.11. Did the participant swallow the three drugs in your presence? 1. Yes 2. No

2.2.12. Did the participant regurgitate the drugs? 1. Yes 2. No

2.2.13. Were the drugs taken again after regurgitating? 1. Yes 2. No

2.2.14. Was the treatment validated? 1. Yes 2. No

(Thank the subject for his participation and keep his file for surveillance)

Appendix 4: AES Surveillance form

3.1. Community ID: |__ |__||___||__ |__||___|

3.2. Participant ID |__ |__||___||__ |__||___||__ |__||___|

3.2.1. Dates of first filling-in for surveillance purposes|_ DD| MM | YY |

3.2.1.1. Which day(s), please underline D1, D2, D3, D4, D5, D6, D7, D8, D9, D10, D11, D12, D13, D14, D15, D16, D17, D18, D19, D20, D21, D22, D23, D25, D26, D27, D28, D29, D20

3.2.2. Place of first filling-in? 1. Survey site 2. Home 3. Other 99. unknown

3.2.3. If other in 4.1.2., specify the place……………………………………………….

3.2.4. Did you take all the three drugs? 1. Yes 2. No 99. unknown

3.2.5. Did you regurgitate the drugs? 1. Yes 2. No 99. Unknown

3.2.6. When did you take the drugs? |_ DD| MM | YY |

3.2.7. How did you feel after taking the drugs? 1. Fine (better than before) 2. Same as before 3. Bad 98. Do not know

3.2.7.1. If the respondent says **fine (better than before) for 4.1.8,** review questions under 2.1 "**For complaints or events prior to the treatment" to describe their evolution.** Use the coding; 0 = **None**: the complaint has gone, 1=**Improved**: the complaint is significantly less than before the treatment, 2 = **Ongoing**: the complaint is the same as before the treatment, 3 = **Exacerbated**: the complaint increased after the treatment; to grade them as in the list below

	Events existing before the treatment	complaints (tick as necessary)	Grade
4.1.8.1.1	Fever		
4.1.8.1.2	Itching/ground itch		
4.1.8.1.3	Headaches		
4.1.8.1.4	Dizziness		
4.1.8.1.5	Tiredness		
4.1.8.1.6	Deafness		
4.1.8.1.7	Jaundice		
4.1.8.1.8	Weakness		
4.1.8.1.9	Fatigue		
4.1.8.1.10	Nausea		
4.1.8.1.11	Vomiting		
4.1.8.1.12	Diarrhoea		
4.1.8.1.13	Abdominal pain		

4.1.8.1.14 Flatulence/dyspepsia

4.1.8.1.15 Constipation

4.1.8.1.16 Joint/muscular pain

4.1.8.1.17 Swelling of (upper/lower) limbs

4.1.8.1.18 Swelling of eyelids/abnormal feeling in the eyes

4.1.8.1.19 Rash/plaque

4.1.8.1.20 Scrotal reaction

4.1.8.1.21 Skin nodules

4.1.8.1.22 Worm expulsion

4.1.8.1.23 Haematuria

4.1.8.1.24 Lymphoedema

4.1.8.1.25 Elephantiasis

4.1.8.1.26 Hydrocele

4.1.8.1.27 Palpitation/tachycardia

4.1.8.1.28 Orthostatic hypotension

4.1.8.1.29 Others

4.1.8.1.30 **If other specify……………………. ……………………………………………….. …………………………………… ………………………………… ……………………………**

3.2.7.2. Did the respondent experience complaints after taking the 3 drugs? 1. Yes 2. No 99. unknown

3.2.7.3. If yes, which one(s)? (See questions under 4.1.8.1)...................................... ....

3.2.7.4. How would you qualify your main complaint (see questions under 4.1.8.2), after taking the 3 drugs **1 = Improved 2 = Ongoing 3 = Exacerbated 4= Resolved**

3.2.7.5. If the respondent says **Bad (worsened than before treatment) for 4.1.8,** review questions under 2.1 "**For complaints or events to prior to the treatment" to describe their evolution.** Use the coding; 0 = **did not feel sick**, 1=**mild**, 2 = **Moderate**, 3 = **Major/severe**; to grade them as in the list below

3.2.7.6.

**Adverse event due to worsened existing symptoms before treatment**			
**Symptoms/signs**	**when did the side effects appeared**	**Evaluation**	**Code**

		D1	D2	D3	D4	D5	D8	D2	D8
4.1.8.5.1	Fever								
4.1.8.5.2	Itching/ground itch								
4.1.8.5.3	Headaches								
4.1.8.5.4	Dizziness								
4.1.8.5.5	Tiredness								
4.1.8.5.6	Deafness								
4.1.8.5.7	Jaundice								
4.1.8.5.8	Weakness								
4.1.8.5.9	Fatigue								
4.1.8.5.10	Nausea								
4.1.8.5.11	Vomiting								
4.1.8.5.12	Diarrhoea								
4.1.8.5.13	Abdominal pain								
4.1.8.5.14	Flatulence/dyspepsia								
4.1.8.2.15	Constipation								
4.1.8.5.16	Joint/muscular pain								
4.1.8.5.17	Swelling of (upper/lower) limbs								
4.1.8.5.18	Swelling of eyelids/abnormal feeling in the eyes								
4.1.8.5.19	Rash/plaque								
4.1.8.5.20	Scrotal reaction								
4.1.8.5.21	Skin nodules								
4.1.8.5.22	Worm expulsion								
4.1.8.5.23	Haematuria								
4.1.8.5.24	Lymphoedema								
4.1.8.5.25	Elephantiasis								
4.1.8.5.26	Hydrocele								
4.1.8.5.27	Palpitation/tachycardia								
4.1.8.5.28	Orthostatic hypotension								
4.1.8.5.29	Others								
4.1.8.5.30	**If other specify……………………**	………………………………………………							

…………………………………… ………………………………… ……………………………

NB: FOR REPORTED SEVERE SIDE EFFECT, COMPLETE THE QUESTIONS UNDER 4.3

3.3. **Adverse events after taking the three drugs be captured in the questions that follows:** defined as; 1 **= mild:** easily tolerated, does not interfere with daily activity, **2 = moderate :** uncomfortable enough to interfere with daily activity, **3 = severe :** precludes daily activity,

	Adverse Events after taking the three drugs																
	**Symptoms/signs**	when did the side effects appeared Evaluation															
		**D1**	**D2**	**D3**	**D4**	**D5**	**D8**	**D2**	**D8**	**D9**	**D10**	**D11**	**D12**	**D13**	**D14**	**D15**	**D30**
4..2.1	Fever																
4.2.2	Itching/ground itch																
4.2.3	Headaches																
4.2.4	Dizziness																
4.2.5	Tiredness																
4.2.6	Deafness																
4.2.7	Jaundice																
4.2.8	Weakness																
4.2.9	Fatigue																
4.2.10	Nausea																
4.2.11	Vomiting																
4.2.12	Diarrhoea																
4.2.13	Abdominal pain																
4.2.14	Flatulence/dyspepsia																
4.215	Constipation																
4.2.16	Joint/muscular pain																
4.2.17	Swelling of (upper/lower) limbs																

4.2.18	Swelling of eyelids/abnormal feeling in the eyes	
4.2.19	Rash/plaque	
4.2.20	Scrotal reaction	
4.2.21	Skin nodules	
4.2.22	Worm expulsion	
4.2.23	Haematuria	
4.2.24	Lymphoedema	
4.2.25	Elephantiasis	
4.2.26	Hydrocele	
4.2.27	Palpitation/tachycardia	
4.2.28	Orthostatic hypotension	
4.2.29	Others	
4.2.30		**If other specify……………………….** ……………………………………………….. ……………………………………. ………………………………… ………………………………

NB: FOR REPORTED SEVERE SIDE EFFECT, COMPLETE THE QUESTIONS UNDER 4.3

Appendix 5: SAEs recording form

1.7. Community ID: |__ |__||___||__ |__||___|

1.8. Participant ID |__ |__||___||__ |__||___||__ |__||___|

3.4. SAEs form between D1 and D15**; use the codes 1 = Severe, 2 = Very severe**

**Severe effects**		**Days when serious side effects occurred**															
		**D0**	**D1**	**D2**	**D3**	**D4**	**D5**	**D6**	**D7**	**D8**	**D9**	**D10**	**D11**	**D12**	**D13**	**D14**	**D15**	**D30**
4.3.1	Angioneurotic oedema/anaphylactic shock																	
4.3.2	Anuresis																	
4.3.3	Seizures																	
4.3.4	Erythema multiforme																	
4.3.5	Epidermal necrolysis																	
4.3.6	Recurrent symptoms																	
4.3.7	Stevens Johnson Syndrome																	
4.3.8	Others																	
4.3.9	If other specify.......................																	
	....																	
	....																	

AFTER COMPLETING QUESTIONS UNDER 4.3 AND 4.2, THE INVESTIGATOR SHOULD ANSWER THE FOLLOWING QUESTION; THE ANSWER WILL BE YES IF ANY SEVERE EFFECT IS WAS REGISTERED

3.5. Did the interviewed participant experience a serious/severe adverse event after taking the 3 drugs? 1. Yes 2. No 99. unknown

3.6. If yes for 4.5, please list down…………………………………………………..

3.7. If yes for 4.5, please complete the Fever and serious adverse events **surveillance form appendix 2.**

4. Causality assessment criteria:

4.1. **Is the serious effect related to the therapy?** 1. Not related 2. Probably 3. Unlikely

4.1.1. What is your Arguments for decision……………………………………………………. ……………………………………………………………………………………………….

4.1.2. Remarks/Observations regarding this serious adverse effect case management………………
……………………………………………………………………………………

NOTE: ***Serious adverse effects will be treated and observed for 24 hours (maximum) by the physicians on the field. If there is no improvement, the subject will be transferred to the most appropriate health care facility***

Thank you for your collaboration

7.1 List of contraindications to look for: 1=Yes, 2=No

**Contraindications**	**1**	**2**
7.1.1. Pregnancy		
7.1.2. Breastfeeding		
7.1.3. Children less than 5 years of age (≤90cm)		
7.1.4. Allergy to one of the study drugs: azithromycin, Ivermectin, Abendazole		
7.1.5. Medicine intake: antiacids containing aluminium or magnesium, nelfinar, ergot derivatives		
7.1.6. Serious illness (systemic edema, severe dyspnea, etc.)		
7.1.7. Others………………………..<br>If others, please specify:		

DECISION REGARDING INCLUSION IN THE STUDY *: 1. Yes, 2. No*

7.2. Concomitant Treatments: codes; 0: Not administered 1: Administered; D=day

Concomitant Treatments	D0	D 1	D2	D3	D4	D5	D6	D7	D8	D9	D10	D11	D12	D13	D14	D15	D30	code
Antalgic treatment: paracetamol																		
Anti-inflammatory treatment: diclofenac																		
Anti-malarial treatment: artemisin and quinine compounds																		
Antibiotics treatment: all categories																		
Local treatment (eyes, lymphoedema, skin, etc.)																		
Solutes																		
Corticoids																		
Cardio-stimulants																		
Anxiolytics																		
Antihistaminics																		

7.3. Concomitant Treatment: Number of days|___|___| days

7.4. Evolution: Favourable: 1. Yes 2. No

Appendix 6: Participant Information Sheet for Co-administration Participants

History of revision		
1	5/16/2018	Conditionally approved by LSHTM, waiting the approval from Ethiopia
2	09/08/2018	Approved by AHRI/ALERT Ethics Committee

Participant Information Sheet for Co-administration Participants

(Read by the HEWs to the participants in the local language)

Title of Project: Safety of the co-administration of azithromycin, albendazole and ivermectin versus standard treatment regimens during mass drug administration (MDA) in Ethiopia: a cluster-randomized trial

Introduction

We would like to invite you to take part in a research study. Joining the study is entirely up to you. Before you decide, you need to understand why the research is being done and what it would involve. One of our team will go through this information sheet with you, and answer any questions you may have. Ask questions if anything you read is not clear or you would like more information. Please feel free to talk to others about the study if you wish. Take time to decide whether or not to take part.

What is the purpose of the study?

The FMOH currently provides three different treatments to your community to keep you safe from trachoma, onchocerciasis. The London School of Hygiene and Tropical Medicine (LSHTM), the Armauer Hauser Research Institute and the Federal Ministry of Health in Ethiopia are trying to discover if three drugs can be given all at once rather than taken two weeks a part as your community is accustomed to.

Why have I been asked to take part?

We have divided up this district randomly so that some people will take ivermectin and albendazole together and then azithromycin two weeks later which is the standard way that your community has been treated in the past and some people will take all three treatments at the same time. Your gote has been randomly placed in the (Participant will be informed which group they are in) group.

Do I have to take part?

No. It is up to you to decide to take part or not. If you don't take part, you will receive the normal treatment according to the standard MDA schedule. Similarly, your children can

Decide if they want to take part and you, as their guardian, can decide if they should be involved in the study.

We will discuss the study together and give you a copy of this information sheet. If you agree to take Part, we will then ask you to sign a consent form.

What will happen to me if I take part?

In both arms you and any children in your guardianship will receive treatments to keep you save from onchocerciasis, lymphatic filariasis, trachoma and soil transmitted helminths. The purpose of the triple MDA is to minimize cost by reducing logistic expenses.

After reading you the consent form, if you agree to participate the following steps will occur: You will first be registered and given a bracelet to wear for the next two weeks which will help us keep track of your information without using a registration card. A nurse will then give you a non-invasive health interview to see if you have any existing health issues. (The next steps depend on which group the participant is in):

> ***If in co administration group:*** *Your height will then be measured to determine the appropriate dosage for each drug. You will be given all three treatments sequentially, taking as much time as you need to swallow the doses. We also ask that you help the HEW administering the drugs measure and dose any children with you. Clinical nurses will then follow-up with you the day after the treatment and ask you basic questions about how you feel. After the second day, you are encouraged to report if you feel ill to your HEW who will inform the clinical nurse.*
>
> ***If in standard group:*** *Your height will then be measured to determine the appropriate dosage for ivermectin/albendazole. A clinical nurse will follow up with you the next day to conduct a simple health interview. After that point, if you feel unwell you are encouraged to inform your communities H.D.A. or HEW who will then inform the clinical nurse stationed in the area. One to two weeks later, the team will return to your community to administer a dose of azithromycin. A clinical nurse will follow up with you the next day to conduct a simple health interview. After that point, if you feel unwell you are encouraged to inform your communities H.D.A. or HEW who will then inform the clinical nurse stationed in the area.*

What are the possible risks and disadvantages?

There could be minimum chance of infection. We will use standard aseptic methods during physical examination and administration of drug to minimize such risk. Also, there is a small chance of temporary illness and/or discomfort related to the drugs. We will follow make sure that all necessary procedures and means are ready to handle any complaint, and minimize risk. In case of the rare SAEs and not possible to handle at the site we will make sure you will be evacuated to appropriate referral centre. All your treatments will follow standard procedures at no cost to yourself.

What are the possible benefits?

The main objective of this work is to reduce const by cutting unnecessary repetitive MDA activities and achieve equivalent effective MDA with less cost.

What if something goes wrong?

If you have a concern about any aspect of this study, you should ask to speak to the researchers who will do their best to answer your questions <contact number>. If you remain unhappy and wish to complain formally, you can do this by contacting <if LSHTM is the sponsor: Patricia Henley at rgio@lshtm.ac.uk or +44 (0) 20 7927 2626>

The London School of Hygiene and Tropical Medicine holds insurance policies which apply to this study. If you experience harm or injury as a result of taking part in this study, you may be eligible to claim compensation.

Can I change my mind about taking part?

Yes. You can withdraw from the study at any time. You just need to tell the study team that you don' want to be in the study anymore. You will still receive the standard treatments for the disease

described at no cost to you. If you withdraw, we will need to use the data collected on you up to your withdrawal.

What will happen to information collected about me?

All information collected about you will be kept private. Only the study staff and authorities who check that the study is being carried out properly will be allowed to look at information about you. Data may be sent to other study staff in London or Addis Ababa but this will be anonymized. This means that any information about you which leaves the hospital/surgery/clinic, will have your name and address removed so that you cannot be recognized.

The clinical nurse will send some details about you to the study team in the district who will store it securely. Your personal details will be kept in a different safe place to the other study information and will be destroyed five years after the study.

At the end of the project, the study data will be archived at AHRI and the LSHTM. The data will be made available to other researchers worldwide for research and to improve medical knowledge and patient care. Your personal information will not be included and there is no way that you can be identified.

What will happen to the results of this study?

The study results will be published in a medical journal so that other public health practitioners can learn from them. Your personal information will not be included in the study report and there is no way that you can be identified from it.

Who is organising and funding this study?

London School of Hygiene & Tropical Medicine is the sponsor for the research and they have full responsibility for the project including the collection, storage and analysis of your data. The study is funded by the International Trachoma Initiative.

Further information and contact details

Thank you for taking time to hear while this information leaflet is read. If you think you will take part in the study please give your verbal assent when the form is read to you or after reading it.

If you would like any further information, please contact [GP/Doctor/healthcare professional or other nominated person] who can answer any questions you may have about the study.

Contact details:

- ✓ **If you have any questions about this study**: you may contact Dr XXX on XXX or the study doctors in _________________________, (will be given on site)

- ✓ **In case of an emergency:** you should return to _______ or call the study coordinator at any time of the day (24 hours). (will be given on site)

- ✓ **If you have a complaint about the study** : you can address it to AHRI/ALERT ethics committee at0118-962183

- ✓ **If you wish to learn what this study showed**: we will have a community feedback session and this will be communicated to the community representative minimum of three months ahead, please

  keep in-touch with your community representative.

Participant assent form

Protocol Title: **Safety of the co-administration of azithromycin, albendazole and ivermectin versus standard treatment regimens during mass drug administration (MDA) in Ethiopia: a cluster-randomized trial**

<u>This form means</u> I can say "No"

I have been read and understood the information sheets attached, and have been given the opportunity to discuss the study and ask questions.

I understand that, if I do not wish to participate in the study I will still receive the standard health care I deserve.

I ………………………………………………… agree to participant in the study and I agree to have regular checkups and report on any discomfort I might experience.

I am aware that I can withdraw my consent at any time of my own choosing or the decision of my physician.

Date | DD____|___MM |___YY |

Oral Consent provided? ………………………………………….

የክለሳ ታሪክ		
1	5/16/2018	በጊዜዊነት በየለንደን የሃይጂንና ትሮፒካል ሜዲስን ት/ቤት የጸደቀ፤ ከኢትዮጲያ በኩል ያለውን የስነምግባር ውሳኔ የሚጠብቅ
2	09/08/2018	በአህሪ/አለርት ኢቲከወስ ኮሚቴ የጸደቀ

የተሳታፊዎች የመረጃ ቅጽ

(በጤና ኤክስቴንሽን ሰራተኞች ለተሳታፊዎች በአማርኛ የሚነበብ)

የጥናቱ ርእስ: *የአዚትሮማሲን፣ አቤንዳዞልና ኢይቨርሜክቲን በእንድላይ በመስጤትና* በተናጠል የመስጤት የማህበረሰብ አቀፍ የመድሃኒት ስርጭት ከጤና አንጻር ያላቸውን ደህንነት ማወዳደር: በግሩፕ (cluster) የሎተሪ ምደባ የሚሰራ ክሊካል ፍተሻ

መግቢያ

ለጥናት እንዲሳተፉ እንጠይቅዎታለን፡፡ በጥናቱ መሳተፍ የግልዎ ውሳኔ ነው፡፡ ከመወሰንዎ በፊት ስለጥናቱ አላማና ምንምን እንደሚያካትት መገንዘብ አለብዎት፡፡ ዐንድ የጥናቱ ቡድን አባል ይህንን ቅጽያነብልዎታል፤ ለሚያነሱዋቸው ጥያቄዎች መልስ ይሰጣል፡፡ ያልገባዎት ነገር ካለ ወይም ተጨማሪ መረጃ ከፈለጉ ማናቸውንም ጥያቄ መጠየቅ ይችላሉ፡፡ አስፈላጊ ሆኖ ከተሰማዎት ስለጥናቱ ከሌሎች ጋር ይወያዩ፡፡ ለመሳተፍ ወይም ላለመሳተፍ ከመወሰንዎ በፊት ጊዜ ወስደው ያስቡ፡፡

የጥናቱ አላማ ምንድን ነው?

የማህበረሰብዎትን የጥገኛ ትላትል፣ ትራኮማና የአይነ ስውርነት ለመጠበቅ የፊደራል ጤና ጥመቃ ሚ/ር.ሶስት የተለያዩ መድሃኒቶችን በተለያየሁኔታ ይሰጣል፡፡ የፌደራል የጤና ጥበቃ ሚ/ር፣ የአርማወር ሃንሰን የምርምር ተቋምና የለንደን የሃይጂንና ትሮፒካል ሜዲስን በመሃበረሰብዎ መድሃኒቶችን በሁለት ሳምንት ልዩነት ሁለተ ጊዜ ከመውሰድ ይልቅ አዲስ የጥቅል በአንድጊዜ የመድሃኒት አሰጣጥ ለመሞከር ታቅዱዋል፡፡

ለምን በዚህ ጥናት እንዲሳተፉ ጋበዝንዎት?

በሎተሪ እጣ አወጣጥ ይህንን ወረዳ ወደ ሁለት ከፍለንዋል፤ አንደኛው ቡድን አይቨር ሜክቲንንና አልቤንዳዞልን ከወሰደ ከሁለት ሳምንት በሁዋላ አዚትሮማክሲንን ይወስዳል ይህ የተለመደው አሰራር ነው፣ በሌላው ቡድን የሚገኙ ሰዎች ሶስቱንም መድሃኒቶች በአንድ ጊዜ ይወስዳሉ፡፡ እርሶ የሚኖሩበት ጎጥም ከሁለቱ በአንዱ ቡድን ተፈርጇል፤ተሳታፊዎች በየትናው ብድን እንደሆኑ ይነገራቸዋል፡፡

መሳተፍ አለብዎትን?

ዓይ፣ መሳተፍ በእርሶ ነጻ ውሳኔ የተመሰረተ ነው፡፡ መሳተፍ ካልፈለጉ በተለመደው አካሄድና ፕሮግራም መድሃኒቶችን ይሰጥዎታል፤ እንዲሁም ልጆችዎት በተመሳሳይ እርሶ እንደቤተሰብ ሃላፊነተዎ ና ልጆችዎ ለመሳታፍ/ላለመሳተፍ ይወስናሉ፡፡ በዚህ ቅጽ ላይ እንወያያለን፤ ቅጹንም መውሰድ ይችላሉ፡፡ ለመሳተፍ ከወሰኑ የስምምነት ቅጽ እንዲፈርሙልን እንጠይቅዎታለን፡፡

ቢሳተፉ ምን ሊያጋጥምዎት ይችላል?

በሁለቱም ቡድን ቢሆኑ እርሶናና ቤተሰብዎን *የጥገኛ ትላትል፣ ትራኮማና የአይነ ስውርነት* ከመጠበቅ መድሃኒቱ ይሰጣጭሁዋል፡፡ ይህ በጥቅል የመስጠት ሂደት የስራ ድግግሞሽን በማስቀረት ወጪ ቆጣቢ ይሆናል፡፡

ይህ ቅጽ ከተነበበልዎት በሁዋላ ለመሳተፍ ከወሰኑ የሚከተሉትን ሂደቶች እንከተላለን፤ እንመዘግብዎትና ለሁለት ሳምንት ለክትትል እንዲመቸን የእጅ አንባር እንዲያደርጉ እንጠይቅዎታለን፡፡ ከዚየም የጥናቱ ነርስ ስለጤናዎ አጠይቅ ይቀርብልዎታል፤ የሚቀጥሉት ሂደቶች ግን እንደየቡድኑ ይለያያሉ፡፡

> ***በጥቅል ሶስት መድሃኒት በአንዴ የሚወሰድበት ቡድን ከሆኑ፤*** *የእያንዳንዱን መድሃኒት መጠን ለመወሰን ቅመትዎን እንለካለን፡፡ ከዚያም ሶስቱንም አይነት መድሃኒቶች በቅደም ተከተል ይወስዳሉ፤ ለመዋጥ የሚፈልጉትን ያህል ጊዜ መውሰድ ይችላሉ፡፡ በተጨማሪም ከርሶ ጋር ያለ ልጅን ለመለካትና መድሃኒቱን ለመመጠን መድሃኒቱን የምታሰራጨውን የጤና ኤክስቴንሽን ሰራተኛ እንዲያግዞዎት እንጠይቅዎታለን፡*

፡ የጥናቱ ነርስ በሚቀጥለው ቀን የክትትል ጥያቄዎችን፣ ምን እንደሚሰማዎት ትጠይቅዎታለች፡፡ ከሁለተኛው ቀን በሁዋላ ጤንነት ካልተሰማዎት ለጤና ኤክስቴንሽን ሰራተኛዋ ሪፖርት እንዲያደርጉ እናበረታታለን፣ እሱዋም የምርምር ነርሱዋ ሪፖርት ታደርጋለች፡፡

በተለመደው የተቀናጀ የማህበረሰብ መድሃኒት ስርጭት ቡድን ከሆኑ፡ *የአልቤንዳዞልና ኢይቨርሜክቲንን መጠን ለመወሰን ቁመትዎን እንለካለን፡. የጥናቱ ነርስ በሚቀጥለው ቀን የክትትል ጥያቄዎችን፣ ምን እንደሚሰማዎት ትጠይቅዎታለች፡፡ ከሁለተኛው ቀን በሁዋላ ጤንነት ካልተሰማዎት ለጤና ኤክስቴንሽን ሰራተኛዋ ሪፖርት እንዲያደርጉ እናበረታታለን፣ እሱዋም የምርምር ነርሱዋ ሪፖርት ታደርጋለች፡፡. ከሁለት ሳምንት በሁዋል የጥናቱ ቡድን አዚትሮማክሲንን ለመስጠት ወደአካባቢያችሁ ይመለሳል፡፡ የጥናቱ ነርስ በሚቀጥለው ቀን የክትትል ጥያቄዎችን፣ ምን እንደሚሰማዎት ትጠይቅዎታለች፡፡ ከሁለተኛው ቀን በሁዋላ ጤንነት ካልተሰማዎት ለጤና ኤክስቴንሽን ሰራተኛዋ ሪፖርት እንዲያደርጉ እናበረታታለን፣ እሱዋም የምርምር ነርሱዋ ሪፖርት ታደርጋለች፡፡*

ሊከሰቱ የሚችሉችሉ እጦቶችና ተጋላጭነት?

በዚህ የጥናት ሂደት አነስተኛ የኢንፌክሽን አጋጣሚ ቢኖርም እስታንዳርድ የሆኑ የንጽህና አግባቦችን ተጠቅመን ምርመራዎችንና የመድሃኒት መስጠት ሂደትን በመከወን ሁኔታውን ለመቀነስ እንጥራለን፡፡ በተጨማሪም ከመድሃኒቶች ባህሪ የተያያዘ መጠነኛ ህመም/የምኞት መጉዋደል ሊከሰት ይችላል፣ ክትትል በማድረግ አስፈላጊ እርዳታ በማረጋገጥ ሊከሰቱ የሚችሉ ተጋላጭነቶችን ለመቀነስ እንጥራለን፡፡ በልዩ አጋጣሚ ሊፈጠር የሚችል አደገኛ የጤና እክል ቢከሰት ወደ ከፍተኛ ህክምና ለማድረስና እንዲረዱ ለማድረግ ዝግጂት ይደረጋል፡፡ ሁሉም የመድሃኒት መስጠትና ተያያዥሂደት እስታንዳርዱን በጠበቀመልኩ ይካሄዳል፡፡

ሊገኙ የሚችሉት ጥቅሞች ምንድናቸው?

ይህ ጥናት በዋናነት የስራ ድግግሞሽን በማስቀረት ወጪ ቆጣቢ በሆነ መንደድ ከዘልማዳዊው የማህበረሰብ አቀፍ የመድሃኒት ስርጭት ተመጣጣኝ ጥቅምን ምስገኘት ነው

መጥፎክስተት ቢከሰት ምን ይደረጋል?

ስለዚህ ጥናት ጥያቄ ካለዎት ጥያቄዎትን ሁሉም አጥኚዎቹ ለመመለስ እንጥራለን፤ የጥናቱ መሪ ተመራማሪ ዶ/ር እንዳላማው ጋዲሳን በ0911868827 ማግኘት ይችላሉ፤ ይህም ካላረካዎት ቅሬታ ካለዎትለአህሪ አለርት የስነምግባር ኮሚቴ በ 0118-962183 ይደውሉ፡፡

የለንደን የሃይጂንና ትሮፒካል ሜዲስን ለዚህ ጥናት ኢንሹራስ ገብቷል፤ በጥናቱ በመሳተፍዎ የደረሰ ጉዳት ካለ ኢንሹራስ የማግኘት መብት አለዎት.

በጥናቱ ስለመሳተፍ ሃሳቤን መቀየር እችላለሁን?

አዎ፣ ከጥናቱ በማንኛውም ግዜ ራስዎን ማግለል ይችላሉ፡፡ እርሶ ለማናኛውም የአጥኙው ቡድን አባል እራስዎን ማግለልዎን መናገር ይችላሉ፡፡ እንደዚህም ሆን ሰታንዳርዱን የተቀናጀ የመድሃኒት ስርጭት ተጠቃሚ ይሆናሉ፡፡ ከጥናቱ እስከወጡበት ቀን ያለውን መረጃ ግን እንጠቀምበታለን፡፡

ከእኔ የተሰበሰበው መረጃ ምን ይደረጋል?

ከእርሶ የተሰበሰበው ሁሉም መረጃ በሚስትር ይያዛል፤ የዚህ አጥኚው ቡድንና የጥናቱን አካሄድ የሚቆጣጠሩ ባለስድርሻዎች ብቻ ይህን መደኛ በቀጥታ ያያሉ፡፡ የጥናቱ መረጃ ለንደንና አዲስ አበባ ላሉ የአጥኝው ቡድን አባላት ሊላክ ይችላል እንዲዚህ ሲሆን መረጃው የመረጃ ሰጭውን ስም አይጠቅስም፡፡ ይህ ማለት ማንኛውም መረጃ ከሆስፒታል/ቀዶ ጥገና/ክሊኒክ ውጭ የርሶን ስም አይዝም፣ ስለሆነም መረጃ ሰጭውን ለይቶ ማወቅ አይቻልም፡፡

የጥናቱ ነርስ ስለእርሶ ውስን መረጃዎችን በወረዳው ለሚገኙ የአጥኝው ቡድን አባላት ይልካል/ትልካለች፡፡ የርሶን ልዩ መግለጫ የያዘው ፋይል ተለይቶ በጥንቃቄ/በሚስትር ይቀመጥና ጥናቱ ከተጠናቀቀ ከአምስት አመት በሁዋላ ይወገዳል፡፡

ጥናቱ ከተጠናቀቀ በሁዋል መረጃዎች በአርማወር ሃንሰን የምርምር ተቋምና በለንደን የሃይጂንና ትሮፒካል ሜዲስን ትምህትቤት ይቀምታል፤ ይህም ለሌሎቸት ተመራማሪዎችና ጤና አጠባበቅን ለማሻሻል ለሚሰሩ እንዲጠቀሙበት ይደረጋል፡፡ ግላዊ መረጃዎች አይቀመጡም፣ ማንም እርሶን ለይቶ ማወቅ አይችልም፡፡

የዚህ ትናት ውጤት ምንደረጋል?

የዚህ ጥናት ውጤት ሌሎች በህክምና ዘርፍ የሚሰሩ ይማሩበት ዘንድ በመጽሄት እንዲታተም ይደረጋል:: በዚህ እትመት የእርሶ ስም አይጠቀስም፣ ማንም ህትመቱን አይቶ ለይቶ ሊያውቅዎት አይችልም፡፡

ይህን ጥናት የሚያቀናጀውና በገንዘብ የሚደግፈው ማነው?

በለንደን የሃይጂንና ትሮፒካል ሜዲስን ትምህት ቤት የዚህ ጥናት ሙሉ ስፖንሰር ነው፤ መረጃ ከማሰባሰብ፣ማጠራቀምና ማጠናቀርን ጨምሮ፡፡ የገንዘብ ድጋፍ ምንጩ የአለማቅ ትራኮማኢኒሼቲቭ ነው፡

ተጨማሪመረጃ ለማግኘት አድራሻዎች

ግዜ ወስደው ይህ መረጃ እስኪነበብልዎ ስለታገሱ እናመሰግናለን፤ ለመሳተፍ ከወሰኑ ለተሳታፊዎች የስምምነት በጠየቂያ ቅጹን ከነበቡ/ከተነበበልዎት በሁዋላ መስማማዎትን ይግለጹ፡፡

ተጨማሪ መረጃ ማግኘት ቢያስፈልግዎት፤ እባክዎትን በቦታው የተመደበውን የጤና ባለሙያ ያነጋግሩ አስፈላጊውን መረጃ ይሰጥዎታል፡፡

ለመረጃ:

✓ **ለማናቸውም ጥናቱን የተመለከተ ጥያቄ**: የጥናቱን የጤና ባለሙያ በ---------------ያገኛሉ (በወቅቱ የሚገለጽ)

✓ **ለአደጋ ጊዜ: የጥናቱን አስተባባሪ በ** ________ በ24 ሰአት ውስጥ ያግኙ (በወቅቱ የሚገለጽ)

ስለትናቱ ቅሬታ ካለዎት ለአህሪ አለርት የስነምግባር ኮሚቴ በ 0118-962183 ይደውሉ

✓ **ስለጥናቱ ውጤት ለማወቅ**: የዚህ ጥናት ውጤት ግብረ መልስ ለማህበረሰቡ ይገለጻል፤ ይህንንም ፕሮግራም ከሶስት ሳምት ቀድምን ስለምናሳውቅ የማህበረሰብዎን መሪዎች ይጠይቁ፡፡

ለተሳታፊዎች የቃል ስምምነት መጠየቂያ ቅፅ

የጥናቱ ርእስ: *የአዚትሮማሲን፣ አቤንዳዞልና ኢይቨርሜክቲን በአንድላይ በመስጤትና* በተናጠል የመስጤት የማህበረሰብ አቀፍ የመድሃኒት ስርጭት ከጤና አንጻር ያላቸውን ደህንነት ማወዳደር: በግሩፕ (cluster) የሎተሪ ምደባ የሚሰራ ክሊካል ፍተሻ

<u>ይህ የስምምነት መግለጫ ቅጽ፣ እምቢ ማለት እንደምችል ያረጋግጣል፤</u>

የጥናቱ መረጃ ተነቦልኛል/አንብቤ ተረድቻለሁ፤ በቂ የውይይትና የጥያቄ ጊዜ ተሰቶኛል።

በጥናቱ ላለመሳተፍ ብወሰን የሚገባኝን የተለመደውን የማህበረሰብ አቀፍ የመድሃኒት ስርጭት መሳተፍ እንደምችል ተገንዝቤአለሁ፡፡

እኔ ………………….በጥናቱ ለመሳተፍና ተከታታ ምርመራ እንዲገረግልኝና ማንኛውንም የምቾት መጉዋደል ሪፖርት ለማድረግ ተስማምቻለሁ፡፡

ጥናቱን በማንኛውም ግዜ በራሴ ወይም በሃኪሜ ውሳኔ ማቋረጥ እንደምችል ተረድቻለሁ።

ለመሳተፍ ተስማምተዋል/አልተስማሙም

ቀን

Appendix 8: Focus group discussion participant information sheet

History of revision		
1	5/16/2018	Conditionally approved by LSHTM, waiting the approval from Ethiopia
2	09/08/2018	Approved by AHRI/ALERT Ethics Committee

Focus Group/Interview participant information sheet: age 18 years or above

Protocol Title: **Safety of the co-administration of azithromycin, albendazole and ivermectin versus standard treatment regimens during mass drug administration (MDA) in Ethiopia: a cluster-randomized trial**

Participant ID: |__ |__||___||__ |__||___||__ |__||___|

Purpose:

You have been invited to participate in a focus group sponsored by the Federal Ministry of Health, AHRI and the London School of Hygiene and Topical Medicine. The purpose of this (focus group)/(personal interview) is to better understand community perceptions surrounding MDA, specifically the co-administration study you have just participated in. Information learned in this focus group will be used to inform the FMOH and the international community about how you feel about combining three drugs into one administration event in comparison to the standard MDA regimen.

Procedure:

A moderator will ask you several questions while facilitating the discussion. The discussion will be audio-recorded and a note-taker will be present. However, your responses will remain confidential, and no names will be included in the final report. You can choose whether or not to participate in the focus group, and you may stop at any time during the course of the study. Please note that there are no right or wrong answers to focus group questions. The facilitator wants to hear the many varying viewpoints and would like for everyone to contribute their thoughts. Out of respect, please refrain from interrupting others if participating in a group exercise. However, feel free to be honest even when your responses counter those of other group members.

Benefits and Risks:

Your participation may benefit you and other communities by determining if co-administration of ivermectin, albendazole and azithromycin is a viable strategy to combat NTDs. However, we anticipate very minor risks of discomfort like those experienced during an average conversation.

Confidentiality:

Should you choose to participate, you will be asked to respect the privacy of other interviewees/ focus group members by not disclosing any content discussed during the study. Researchers within LSHTM will analyze the data, but—as stated above—your responses will remain confidential, and no names will be included in any reports.

Contact Dr Endalamaw Gadisa the Ethiopian PI if you have any questions with 0911868827 or if you have any concerns regarding this study, please contact the AHRI/ALERT ethics bureau with 0118-962183 :

Focus Group/Interview consent form: age 18 years or above

<u>This form means</u> I can say "No"

I have been read and understood the information sheets attached, and have been given the opportunity to discuss the study and ask questions.

I am aware that I can withdraw my consent at any time of my own choosing.

1. I ………………………………………………… to be enrolled
   a. agree b. Disagree
2. I ……………………..to respect other participants right and keep all information I get from this discussion confidential.
   a. agree b. Disagree

Date | DD___ |___MM |___ YY |

Sign ……………………………………………..

የክለሳ ታሪክ		
1	5/16/2018	በጊዜዊነት በየለንደን የሃይጂንና ትሮፒካል ሜዲስን ት/ቤት የጸደቀ፤ ከኢትዮጲያ በኩል ያለውን የስነምግባር ውሳኔ የሚጠብቅ
2	09/08/2010	በአህሪ/አለርት ኢቲክስ ኮሚቴ የጸደቀ

የተመረጡ ግለሰቦች ውይይት (Focus Group) ለመሳተፍ የመረጃ፤ 18 ዓመትና ከዚያ በላይ ለሆናቸው

የጥናቱ ርእስ: *የአዚትሮማሲን፣ አቤንዳዞልና ኢይቨርሜክቲን በአንድላይ በመስጤትና* በተናጠል የመስጤት የማህበረሰብ አቀፍ የመድሃኒት ስርጭት ከጤና አንጻር ያላቸውን ደህንነት ማወዳደር: በግሩፕ (cluster) የሎተሪ ምደባ የሚሰራ ክሊካል ፍተሻ

<u>ይህ የስምምነት መግለጫ ቅጽ፣ እምቢ ማለት እንደምችል ያረጋግጣል፤</u>

የጥናቱ አላማ

እርሶ በፌደራል የጤና ጥበቃ ሚ/ር፣ በአርማወር ሃንሰን የምርምር ተቋምና በለንደን የሃይጂንና ትሮፒካል ሜዲስን ቅንጂት የሚሰራ ጥናት እንዲሳተፉ ተጋብዘዋል፡፡ የዘህ ከተመረጡ ግለሰቦች ጋር የሚደረግ ውይየት አላማ በጥቅል ሶስት መድሃኒቶችን በመስት የሚደረግ ማህበረሰብዎን ለተቀናጀ ማህበረሰብ አቀፍ የመድሃኒት ስርጭት አተያየት ለመገምገም ነው፡፡ ስለ ጥቅልና የተለመደው በተናጥል መድሃኒቶችን በመስጠት የሚካሄድ የተቀናጀ የመድሃኒት ስርጭት በማወዳደር ያለዎትን አተያይ በማጠናቀር የሚገኘውን መረጃ ለኢትዮጲያ የፌደራል ጤናጥበቃንና የአለማቀፍ ማህበረሰብን ለማሳወቅ ነው፡፡

አካሄድ:

የውይይቱ አስተባባሪ የውይይቱን ሂደት በማቀናጀት በርካታ ጥያቄዎችን ይሰነዝራል፡፡ ውይይቱ በድምጽ መቅረጫ ይቀዳል፤ ሂደቱንም በማስታወሻ የሚይዝ ሰው አብሮ ይኖራል፡፡ ሑሉም መረጃ በሚስጥር የሚያዝ ይሆናል፤ የርሶ ስም መሪፖርት ላይ አይነገርም/አይቀመጥም፡፡ በውይይቱ ላለመሳተፍ ሊወስኑ ይችላሉ፤ በማንኛውም የጥናቱ ሂደት ማቋረጥም ይችላሉ፡፡ ልብ ይበሉ! በዚህ ውይይት ለሚሰነዘሩ ጥያቄዎች ትክክል/የተሳሳተ የሚባል መልስ የለም፡፡ አወያዩ የተለያዩ አተያዮችን መስማትና እያንዳነዱ ተሳታፊ የየራሱን እንዲያዋጣ ይፈልጋል፡፡ ካለመከባበር፣ እባክዎትን በቡድን ውይይቱ ሌሎችን ከማቋዋረጥ ይቆጠቡ፡፡ ነግር ግን ሃሳብዎ ከሌሎች በተጻራሪ ቢሆንም በሃቀኝነት ሃሳብዎን ይናገሩ::

ጥቅምና ተጋላጭነት:

የእርሶ መሳተፍ ለእርሶና ለማህበረሰብዎ *የአዚትሮማሲን፣ አቤንዳዞልና ኢይቨርሜክቲን በእንድላይ የመስጠት* የማህበረሰብ አቀፍ የመድሃኒት ስርጭት ያለውን አተያይ እንድናውቅ ስለሚረዳን ይህ መድሃኒቶቹን በጥቅል የመስጠት አሰራር ትኩረት የሚሹ የቆላና ሃሩር በሽታወችን ለመመከት ተገቢነት ያለው መሆኑን ለመወሰን ይረዳል፡፡ እንደማንኛውም የግሩፕ ውይይት መጠነኛ የስሜት አለመመቻቸት ሊከሰት እንደሚችል እንገምታለን፡፡

ሚስጥር ስለመጠበቅ

በዚህ ውይይት ለመሳተፍ ከወሰኑ፣ የሌሎች ተሳታፊዎችን ግላዊ እራሰን የመግለጽ በብጽ በማክበርና ሌሎችን ኃሳብ ሚስጥር ለመጠበቅ እንዲስማሙ እንጠይቅዎታለን፡፡ የለንደን የሃይጂንና ትሮፒካል ህክምና ትምህርት ቤት ከዚህ ውይይት የሚገኘውን መረጃ ያጠናቅራል፤

ከርሶ የተገኘው መረጃ ከላይ እንደተገለጸው በሚስጥር የሚያዝ ይሆናል፣ እናም በሪፖርት ላይ ስም የማይጠቀስ መሆኑን ደግመን እናረጋግጣለን፡፡

ስለዚህ ጥናት ጥያቄ ካለዎት የጥናቱ መሪ ተመራማሪ የሆኑትን ዶ/ር እንዳላማው ጋዲሳን በሚከተሉት ስልኮች ማግኘት ይችላሉ፤

- ዶ/ር እንዳላማው ጋዲሳ 0911868827

✓ ስለትናቱ ቅሬታ ካለዎት ለአህሪ አለርት የስነምግባር ኮሚቴ በ 0118-962183 ይደውሉ፡፡

የተመረጡ ግለሰቦች ውይይት (Focus Group) ለመሳተፍ የስምምነት መግለጫ ቅጽ፤ 18 ዓመትና ከዚያ በላይ ለሆናቸው

የተሳታፊ ኮድ: |__ |__||___||__ |__||___||__ |__||___|

ይህ ቅጽ እንቢ ማለትም እንደሚቻል ያረጋግጣል

የጥናቱ መረጃ ተነቦልኛል/አንብቤ ተረድቻለሁ፤ በቂ የውይይትና የጥያቄ ጊዜ ተሰቶኛል።

ጥናቱን በማንኛውም ግዜ ማቋረጥ እንደምችል ተረድቻለሁ።

1. እኔ ከላይ በተገለጸው ጥናት ለመሳተፍ ተስማምቻለሁ።

   ሀ. ተስማምቻለሁ ለ. አልተስማማሁም

2. ዚህ ውይይት የሚነሱ ማናቸውንም መረጃዎች ሚስጥር ለመጠበቅ፤ በውይይቱ ሂደት የሌሎችን መብት ለማክበር

   ሀ.ተስማምቻለሁ ለ.አልተስማማሁም

ቀን | DD____|___MM |___ YY |

ፊርማ

Appendix 10: Charter of Data and Safety Monitoring Board (DSMB)

This charter is for the DSMB for the "Comparison of the co-administration of azithromycin, albendazole and ivermectin versus standard treatment regimens during mass drug administration (MDA) in Ethiopia: a cluster-randomized trial**."** This study will be conducted in Ethiopia.

The Charter will define the Terms of Reference (ToR) for the DSMB, its membership, its relationship with other trial components, and the purpose and timing of its meetings. The Charter will also provide the procedures for ensuring confidentiality and proper communication, the monitoring guidelines to be implemented by the DSMB, and outline of the content of the Reports that will be provided to the DSMB.

Terms of Reference (ToR)

1. The DSMB for the Co-administration trials are charged with the safety and well-being of the study participants. They also serve as advisors on maintaining data quality and unanticipated study events.

2. The study will generate real time analysis of the data collected and reports including rates and confidence intervals (See **Section 8.9**). As a safety measure, the study leaders will convene an immediate teleconference with the DSMB given the following criteria*:*

- IF the rate of adverse events ranked as mild/moderate by participants differs significantly in the trial group as compared to the control group during the first two "Follow-up Days" **(Day 2, Day 4)** as well during subsequent review of the data at **Day 15**, and at the conclusion of the study on **Day 31** (to determine if the remainder of the woreda can safely be treated via triple drug therapy).

- IF an SAE occurs all related data will be provided to the DSMB members who will make an initial decision about attribution and whether a formal DSMB meeting is required. (Note: Given that a "severe" adverse event includes inability to complete daily activities, it will be classified as a possible SAE for the purposes of this study.)

- At conclusion of the study on **Day 31** (to determine if the remainder of the woreda can safely be treated via triple drug therapy).

3. If the DSMB concludes that there is evidence that warrants stopping the trial or a change in the procedures of the clinical trial, the Board will be responsible for drafting a report of their recommendations to the Principal Investigators and to the IRB of the LSHTM and AHRI. If there is no concern, the Board will also issue a report stating they have no concerns.

4. The DSMB shall meet by phone at Day 31 regardless of the occurrence of AEs and SAEs for the purposes of reviewing the accumulated data, the quality of the information collected the safety of the trial procedures and the efficacy of the treatment arms. The committee shall issue a summary report, which will be forwarded to the Co-administration study PIs, the Federal Ministry of Health of Ethiopia, and the LSHTM and AHRI IRB.

5. The location of the trial is in rural Africa. Social, cultural, or political exigencies may arise that may affect the trial or call into question some of the protocols of the trial. The DSMB has accepted the responsibility to serve as a review body for such matters, if requested by members of the DSMB or members of the study team. The members of the DSMB may request

a review by the full committee of any events arising from the study which, in the opinion of the committee member, is a serious issue or one in which a response to a government may be necessary. A conference call would be organized as expeditiously as possible with a quorum of members. For this purpose, a quorum is considered three of the five members. The call must include the Principal Investigator and the relevant study director. The committee will recommend a course of action to the Principal Investigator regarding any such events. If the study leadership does not concur with the DSMB's recommendation, it will be the responsibility of the study leadership and DSMB Chair to reach a mutually acceptable decision. A minority report will be issued from this group.

DSMB Key Responsibilities

The DSMB will be responsible for carrying out the following responsibilities:

1. Assessing data quality, including completeness.
2. Monitoring reports of recruitment and losses to follow-up.
3. Monitoring reports of the compliance with the treatment protocol by communities and investigators.
4. Monitoring evidence for intervention differences in the main outcomes.
5. Monitoring the safety of interventions (e.g., adverse events).
6. Deciding whether to recommend that the trial continues as planned or whether treatment should be terminated at the time of the interim analysis.
7. Reviewing proposed major modifications to the study prior to their implementation (e.g., increasing target sample size).
8. Suggesting additional data analyses if deemed ethically necessary.
9. Reviewing the interim stopping rule analysis prepared by the study, and communicating the result of that analysis to the PI and executive committee.

DSMB Membership

The DSMB is an independent multidisciplinary group consisting of the following five voting members:

Dr. Liya Wasie (ethics spacialist)
Dr. Dawit Asmamaw (pharmacologist, with DSMB experience)
Mr. Tsegaye Hailu (Biostatistician)
Dr. Eric Ottisen (Task Force member for Global Health)
Dr. Esmael Ali, and
Dr. Christopher King

Conflicts of Interest

The DSMB membership is restricted to individuals free of apparent significant conflicts of interest. The source of these conflicts may be financial, scientific or regulatory in nature. Thus, DSMC members may not be involved in the Co-administration trial, have a vested interest in its outcome, have close personal or professional ties to a Co-administration trial investigator, or have financial investments in any of the pharmaceutical companies donating the drug. If at any time a DSMB member perceives that he/she or another member of the Board has a potential conflict of interest, he/she is obligated to bring the issue to the attention of the full DSMB for open discussion and resolution. Any DSMB members who develop significant conflicts of interest during the course of trial should resign from the DSMB. A replacement will be considered.

DSMB members will complete a conflict of interest disclosure form in advance of the first meeting. Competing interests should be disclosed by all DSMC members. The completed forms will be reviewed by the DSMB Chair. If a significant conflict of interest is noted, the DSMB Chair will determine if a member has a conflict of interest that would compromise their ability to serve on the DSMB. If he recommends resignation, the member will be replaced.

The DSMB membership is to be constituted for the duration of the Co-Administration trial. Before the publication of Co-Administration trial primary results, the DSMB members should not discuss issues from their involvement in the trial.

DSMB Meetings

Precluding an occurrence of adverse events at previously described percentage or an SAE, the DSMB will meet via phone conference at the start of the project, and at Day 31 of the trial. Additional DSMB teleconferences will be scheduled as the Chair recommends. The agenda will be prepared by the DSMB Chair in consultation with the study investigators.

At least five business days before the DSMB the Principal Investigator will send study materials to the DSMB members to allow time to review the material before the meeting. The Principal Investigator and team will be responsible for the preparation of meeting materials and all DSMB meeting arrangements.

Closed sessions of the DSMB will be attended only by the voting DSMC members, the Executive committee, and the study statisticians. The closed session may be called when the DSMC reviews the safety and impact data unmasked as to random assignment. At the discretion of the DSMC Chair, the committee can meet in Executive session with only the five voting members present to discuss issues and take formal votes as needed.

Meeting minutes will be prepared by the Chief Investigator for the trial. Draft minutes will be combined and distributed to the Chair for review and changes before distribution to the entire committee and study team members. The minutes shall summarize all recommendations.

DSMB members must make every effort to attend DSMB meetings. If a member fails to make a meeting, it should be ensured that the member makes the next meeting. If a member fails to make the second meeting, the member will be replaced.

All data presented to the DSMB is considered confidential, and cannot be revealed to those outside the study until after publication.

Protocol Changes and Ancillary Studies

The DSMB will be informed of any major study protocol changes, and DSMC concurrence will be sought on substantive recommendations for changes prior to their implementation. Ancillary studies affecting the trial will be brought forward to the DSMC for their information. The DSMC will review them for possible impact on the main trials. The DSMC will not make recommendations regarding whether the studies may or may not proceed, which is the province of the Executive committee.

Recommendations

DSMB recommendations will be provided to the study leadership. If the recommendations involve participant safety, the Principal Investigator, in collaboration with the site investigators, will act to implement the change as expeditiously as possible. However, if the study leadership does not concur with the DSMB recommendations, it will be the responsibility of the study leadership and DSMB Chair to reach a mutually acceptable decision for implementation, and a report issued to the DSMB.

Annex 11: FOCUS GROUP DISCUSSION GUIDE (Community Volunteers)

TITLE: Safety of the co-administration of Azithromycin, Albendazole and Ivermectin versus standard treatment regimens during mass drug administration (MDA) in Ethiopia: a cluster randomized trial

Greetings,

My name is …………………and am a public health Professional and a PhD student at LSHTM/AHRI. We are here on behalf of AHRI, LTMH and the federal and Oromia health bureaus to invite you to be part of a focus group discussion to capture information on MDA and the triple co-administration MDA for (LF, Trachoma, and STH) control programme in your community.

The information to be gathered would inform us on the perception and level of acceptability of the triple co-administration MDA as compared to the regular MDA that used to be done in your community. If you agree to take-part; you would be requested to meet with other participants for a group discussion on the designated issues in accordance with your knowledge of the MDA and your reaction towards the Triple-co administration MDA. The discussion WOULD NOT LAST 1 HOUR, would be recorded, transcribed and translated into English Language for further analysis. Names would not be mentioned or stated in the transcripts and therefore whatever responses provided would not have any link to the respondent. Only the research team would have the transcripts.

PARTICIPATION IS FULLY VOLUNTARY, at your absolute will, and you can withdraw at any point in time during or after the discussion; *REFUSAL TO PARTICIPATE WOULD NOT AFFECT YOU IN ANY WAY.*

I. **Scociodemographic characteristics**

a. Age _____|_____|_____ Years
b. Sex (underline) Male Female
c. Marital status (underline) Single, Married, Widowed, Divorced
d. Education (underline)
e. Occupation (social responsibility)

II. **Awareness of drug distribution**

1. Has there been any mass drug distribution in this community recently? (Probe further on how long ago was this done? What was the purpose of the drug?
2. How did you get to know about the distribution exercise (probe further on adequacy of information provided and the person who did the distribution)

III. **Process of drug distribution**

3. How was the distribution done? (Probe for Pre distribution exercises, time of the day and feasibility Strategy used and feasibility)
4. In your opinion did everyone in the community receive the drugs? (probe further on category of people who received the drug and those exempted)

IV. **Opinion about drug distribution**

5. Which arm of the distribution was your community (triple co-administration or the standard MDA)
6. Were you satisfied with the present way of drug distribution? (probe further how the current MDA approach differed from previous or current standard MDA)
7. What suggestions do you have for improving future distribution exercises (probe further pill number (burden), strategy, timing, distributors)

V. **Community factors**

8. What is the community/your perceptions towards the types of MDA, triple-co administration vs standard (probe further on number of complaints, logistical, socio-cultural, religious factors).

VI. **MDA related Factors**

9. What are the triple co-administration related challenges? How do community, family and/or close associate relate any complaint to the approach? (probe further on the nature of the complaints, duration and number)

REFERENCES (END NOTES)

[1] Author's note: Much of the information in the protocol is drawn from the FY17 ENVISION Ethiopia Work Plan supported by USAID, written by Scott McPherson. The work plan was written in consultation with the Federal Ministry of Health NTD case team using the National Integrated NTD database (WHO-endorsed tool), the Tool for Integrated Planning and Costing (WHO-endorsed tool), and various NTD implementing partner mappings conducted by the FMOH, RTI International, and others.

[2] Fenwick A. The global burden of neglected tropical diseases. Public Health. 2012; 126: 233–236.

[3] WHO Library: Lymphatic Filariasis: managing morbidity and preventing disability: an aide-memoire for national programme managers
http://apps.who.int/iris/bitstream/10665/85347/1/9789241505291_eng.pdf?ua=1

[4] Gyapong, John, Gyapong, Margaret and Sam Adjei. The Epidemiology of Acute Adenolymphangitis Due to Lymphatic Filariasis in Northern Ghana. Am J Trop Med Hyg June 1996 54:591-595

[5] P. Rebollo M, Sime H, Assefa A, Cano J, Deribe K, Gonzalez-Escalada A, et al. (2015) Shrinking the Lymphatic Filariasis Map of Ethiopia: Reassessing the Population at Risk through Nationwide Mapping. PLoS Negl Trop Dis 9(11): e0004172. doi:10.1371/journal.pntd.0004172

[6] Second Edition of National Neglected Tropical Disease Master Plan, Federal Ministry of Health of Ethiopia: Addis Ababa 2016. https://www.medbox.org/national-neglected-tropical-diseases...plan/download.pdf

[7] Zouré, H. G. M., Wanji, S., Noma, M., Amazigo, U. V., Diggle, P. J., Tekle, A. H., & Remme, J. H. F. The Geographic Distribution of *Loa loa* in Africa: Results of Large-Scale Implementation of the Rapid Assessment Procedure for Loiasis (RAPLOA) 2011. *PLoS Neglected Tropical Diseases*, *5*(6), e1210.

[8] Burnham, Gilbert Onchocerciasis: Review. (1998) The Lancet, Volume 351, Issue 9112, 1341-1346

[9] Onchocerciasis: Guidelines for Stopping Mass Drug Administration and Verifying Elimination of Human Onchocerciasis. WHO Library Cataloguing–in-Publication Data:
http://apps.who.int/iris/bitstream/10665/204180/1/9789241510011_eng.pdf?ua=1

[10] Guidelines for Onchocerciasis Elimination in Ethiopia, Federal Ministry of Health of Ethiopia. Addis Ababa2016:
https://www.cartercenter.org/resources/pdfs/news/health_publications/river_blindness/onchocerciasis-elimination-certification-guidelines-ethiopia.pdf

[11] Zouré, H. G. M., Noma, M., Tekle, A. H., Amazigo, U. V., Diggle, P. J., & Remme, J. H. F. The geographic distribution of onchocerciasis in the 20 participating countries of the African Programme for Onchocerciasis Control: (2) pre-control endemicity levels and estimated number of infected *Parasites and Vectors 2014.* 7:326

[12] Geneva: WHO; 2012. World Health Organization (WHO). *Eliminating soil transmitted helminthiases as a public health problem in children. Progress Report 2001-2010 and Strategic Plan 2011-2020*.

[13] Pullan, R., Smith, J., Rashmi, J. and Brooker, S. Global Numbers of Infection and Disease Burden of Soil transmitted helminth infections in 2010. *Parasites and Vectors* 2014 7:37

[14] Resnikoff S, Pascolini D, Etya'ale D, et al. Global data on visual impairment in the year 2002. Bull World Health Organ 2004; 82:844–51

[15] Solomon AW, Holland MJ, Alexander ND, et al. Mass treatment with single-dose azithromycin for trachoma. N Engl J Med 2004; 351: 1962–71.

[16] Peters, D.H., Friedel, H.A. & McTavish, D. Drugs. 1992 44: 750.

[17] Trachoma Guidelines for Program Managers (WHO) http://apps.who.int/iris/bitstream/10665/43405/1/9241546905_eng.pdf (Last accessed: February 12, 2016)

[18] Mackenzie C.D., Geary T.G., Gerlach J.A. Possible pathogenic pathways in the adverse clinical events seen following ivermectin administrations in onchocerciasis patients. 2003 Filaria J. 2 (Suppl. 1), S5.

[19] Rump, A., & Ōura, S. Ivermectin, "Wonder drug" from Japan: the human use perspective. 2011 *Proceedings of the Japan Academy. Series B, Physical and Biological Sciences*, *87*(2), 13–28.

[20] WHO: Preventive chemotherapy in human helminthiasis http://apps.who.int/iris/bitstream/10665/43545/1/9241547103_eng.pdf

[21] Oxberry ME, Thompson RC, Reynoldson JA Evaluation of the effects of albendazole and metronidazole on the ultrastructure of Giardia duodenalis, Trichomonas vaginalis and Spironucleus muris using transmission electron microscopy. 1994 Int J Parasitol. 24(5): 695-703

[22] Awadzi K, Edwards G, Duke BOL, Opoku NO, Attah SK, Addy ET, Ardrey AE, Quartey BT. The co-administration of ivermectin and albendazole—safety, pharmacokinetics and efficacy against Onchocerca volvulus. 2003 Ann Trop Med Parasitol 97: 165–178.

[23]Guzzo CA, Furtek CI, Porras AG, Chen C, Tipping R, Clineschmidt CM, Sciberras DG, Hsieh JY-K, Lasseter KC. Safety, tolerability, and pharmacokinetics of escalating high doses of ivermectin in healthy adult subjects. 2002. *J Clin Pharmacol* **42***:* 1122–1133

[24] El-Tahtawy A, Glue P, Andrews EN, Mardekian J, Amsden GW, et al. The Effect of Azithromycin on Ivermectin Pharmacokinetics—A Population Pharmacokinetic Model Analysis. 2008. PLOS Neglected Tropical Diseases 2(5)

[25] Coulibaly YI, Dicko I, Keita M, Keita MM, Doumbia M, et al. A Cluster Randomized Study of The Safety of Integrated Treatment of Trachoma and Lymphatic Filariasis in Children and Adults in Sikasso, Mali. (2013) PLOS Neglected Tropical Diseases 7(5): e2221.

[26] Personal communication with the author of the study (Dr. Michael Marks) as results are not yet published

[27] Drawn from presentation made by Columbian FMOH at the Global Elimination of Trachoma (GET2020) meeting in 2015 as well as from direct communication between the Ethiopian FMOH personnel and the Columbian FMOH personnel.

[28] WHO: Preventive chemotherapy in human helminthiasis

http://apps.who.int/iris/bitstream/10665/43545/1/9241547103_eng.pdf

[29] WHO: Trachoma Control- A guide for trachoma program managers
http://apps.who.int/iris/bitstream/10665/43405/1/9241546905_eng.pdf

[30] Bailey RL[1], Arullendran P, Whittle HC, Mabey DC. Randomised controlled trial of single-dose azithromycin in treatment of trachoma. Lancet. 1993 Aug 21;342(8869):453-6

[31] Coulibaly YI, Dicko I, Keita M, Keita MM, Doumbia M, et al. A Cluster Randomized Study of The Safety of Integrated Treatment of Trachoma and Lymphatic Filariasis in Children and Adults in Sikasso, Mali. (2013) PLOS Neglected Tropical Diseases 7(5): e2221.

[32] Martin DH, Mroczkowski TF, Dalu ZA, McCarty J, Jones RB, et al. (1992) A controlled trial of a single dose of azithromycin for the treatment of chlamydial urethritis and cervicitis. The Azithromycin for Chlamydial Infections Study Group. N Engl J Med 327: 921–925.

[33] Ayele B, Gebre T, House JI, et al. Adverse Events after Mass Azithromycin Treatments for Trachoma in Ethiopia. *The American Journal of Tropical Medicine and Hygiene*. 2011;85(2):291-294. doi:10.4269/ajtmh.2011.11-0056.

[34] http://labeling.pfizer.com/ShowLabeling.aspx?id=511

[35] Simonsen P, Magesa S, Dunyo S, Malecela-Lazaro M, The effect of single dose ivermectin alone or in combination with albendazole on Wuchereria bancrofti infection in primary school children in Tanzania.
Trans R Soc Trop Med Hyg (2004) 98 (8): 462-472.

[36] A Handbook for Managing Adverse Events Following Mass Drug Administration (AEs-f-MDA): http://www.ntdenvision.org/resource/other_guidance/sae_handbook_a_handbook_for_managing_adverse_events_following_mass_drug

[37] Adverse drug reactions: definitions, diagnosis, and management. I Ralph Edwards, Jeffrey K Aronson - Lancet 2000; 356: 1255–59
